# Resonance Frequency Analysis and Clinical Outcomes in Implant Dentistry: A Systematic Review and Meta‐Analysis

**DOI:** 10.1111/cid.70156

**Published:** 2026-05-12

**Authors:** Angela Tisci, Francesco Fanelli, Vito Carlo Alberto Caponio, Khrystyna Zhurakivska, Mario Dioguardi, Giuseppe Troiano

**Affiliations:** ^1^ Department of Clinical and Experimental Medicine University of Foggia Foggia Italy; ^2^ Department of Life Sciences, Health and Healthcare Professions Link Campus University Rome Italy; ^3^ Department of Medicine and Surgery LUM University Casamassima Italy

**Keywords:** alveolar bone loss, dental implants, osseointegration, survival rate, torque

## Abstract

**Objectives:**

Given the growing use of resonance frequency analysis in clinical implantology and the inconsistent evidence regarding its prognostic value, this systematic review aimed to clarify the association between implant stability quotient (ISQ) with insertion torque (IT), marginal bone loss (MBL), and implant survival/success rate.

**Material and Methods:**

Following PRISMA guidelines, a comprehensive literature search was conducted across MEDLINE, Scopus, and Web of Science to answer the PIO question: “In systemically healthy patients undergoing implant placement, are ISQ values associated with intraoperative and postoperative surrogate parameters (IT and MBL) and clinical outcomes such as implant survival and success?” Risk of bias was assessed using design‐specific methodological tools.

**Results:**

Forty‐eight studies were included, and 20 were eligible for quantitative analysis of ISQ‐IT correlation. A moderate and statistically significant association was observed (pooled *r* = 0.44; 95% CI: 0.32–0.55, *p* < 0.001), with substantial heterogeneity (*I* ^2^ > 90%). Three studies assessed baseline ISQ values and implant survival. Although surviving implants showed higher ISQ values (mean difference = 10.22), the pooled estimate was not statistically significant and remained highly heterogeneous. Quantitative synthesis for MBL and implant success was not feasible due to inconsistent reporting and methodological variability.

**Conclusions:**

ISQ shows a moderate correlation with IT, supporting its role as a complementary indicator of primary implant stability. However, high heterogeneity and limited certainty of evidence restrict its clinical interpretability. Current evidence does not support baseline ISQ values as independent predictors of MBL or implant survival.

## 1 Introduction

One of the most important prerequisites for the immediate and long‐term clinical functioning of an implant is the formation of a solid implant/bone interface [1]. At any given time point, implant stability represents a unified clinical parameter reflecting the combined effects of mechanical engagement and biological integration. The conventional distinction between primary and secondary stability serves as a conceptual framework: primary stability denotes the mechanical interlocking between the implant and host bone at placement, whereas secondary stability refers to the biologically mediated osseointegration that develops over time. However, in clinical reality, these components are not separate entities but contribute dynamically and concurrently to the overall implant stability [2].

The bone healing process following implant placement involves a cascade of coordinated events, including clot formation, angiogenesis, recruitment of osteoprogenitor cells, and the deposition of peri‐implant woven bone, which subsequently undergoes remodeling into mature lamellar bone [3].

Immediately following implant placement, implant stability is entirely attributable to primary mechanical engagement with the host bone. This initial mechanical stability progressively declines over the first 2 ‐ 3 weeks, primarily due to localized bone resorption induced by the inflammatory response to surgical trauma [4]. Concurrently, the biological healing cascade initiates the formation of new bone at the implant interface, progressively contributing to the restoration of stability. Biological stabilization typically reaches a functional plateau between weeks 5 and 6, coinciding with the completion of early osseointegration [2]. Achieving sufficient primary stability at placement is essential to compensate for the temporary decline in mechanical support during early healing. This ensures that overall implant stability remains above the critical threshold needed to prevent micromovements at the bone–implant interface, which can compromise osseointegration by disrupting osteogenic cell differentiation and promoting fibrous tissue encapsulation [5, 6].

The clinical assessment of successful osseointegration is primarily based on the absence of implant mobility. This evaluation often includes measurements of mechanical stability, such as resonance frequency analysis (RFA), an objective and quantitative method, alongside less standardized and operator‐dependent techniques like manual or mechanical percussion testing or the reverse torque test [7, 8, 9]. The use of stability assessment tools is not limited to research applications; their role is also crucial in clinical prognostic evaluation by supporting the development of individualized loading plans and providing quantitative estimates of implant stability variations over time [1, 2, 10]. Among the noninvasive techniques available for assessing implant primary stability, the most widely used are Insertion Torque (IT) measurement and RFA [11]. IT quantifies the rotational resistance encountered by the implant as it advances apically along its axis, reflecting the frictional interaction with the surrounding bone [12]. Beyond its primary role during implant placement, IT may also provide indirect insights into local bone density [5, 13]. However, this method can only be used at the moment of implant insertion and, due to its non‐repeatable nature, offers no utility for monitoring stability over time. On this basis, modern clinical practice is also based on the RFA for the evaluation of both primary and secondary implant stability. This technique is based on the measurement of the vibrational response of an implant to a magnetic stimulus, which reflects the stiffness of the bone‐implant interface [8]. The recorded resonance frequency values (in Hertz) are then converted into the Implant Stability Quotient (ISQ), a numerical scale ranging from 1 to 100, where higher values indicate greater mechanical stability. The ISQ value is regarded as a helpful indication for tracking changes in implant stability over time, particularly during the osseointegration phase, as it has been demonstrated to correlate with the degree of bone‐to‐implant [2, 14, 15]. Currently, two main commercial systems are available for RFA‐based measurements: Osstell and PenguinRFA with their respective sensors. Osstell uses a single‐use aluminum SmartPeg, while PenguinRFA employs a reusable titanium MulTipeg, which can be sterilized up to 20 times. In both systems, the transducer is screwed to the implant with a defined torque (5 Ncm) and excited by magnetic pulses. RFA is repeatable, noninvasive, and allows for multidirectional assessment of implant fixation, making it a valuable tool in both clinical decision‐making and longitudinal follow‐up [2, 14, 15].

The true predictive ability of RFA and ISQ in reflecting clinically significant parameters, both intraoperatively and postoperatively, remains somewhat uncertain despite their extended clinical use. The existing literature reveals substantial heterogeneity in both techniques and reported clinical outcomes, thereby complicating data interpretation and reducing its translational value in clinical practice. While numerous studies have explored the relationship between RFA and both surrogate parameters, such as IT and marginal bone loss (MBL) and clinical outcomes (implant survival/success), there is still no unified agreement on the strength of these correlations or on the precise clinical indications for RFA implementation. For this reason, the objective of the present systematic review and meta‐analysis was to quantitatively and qualitatively synthesize the available evidence on the association between ISQ values and intraoperative and postoperative parameters, including IT, MBL, and implant survival and success.

## 2 Materials and Methods

Registration of this systematic review and meta‐analysis was performed in the PROSPERO database (Registration no.: CRD42025648858). This study was reported according to Preferred Reporting Items for Systematic Reviews and Meta‐Analyses (PRISMA) guidelines [16].

### 2.1 Search Strategy and Database Screening

A literature search was performed in the MEDLINE database (accessed via PubMed), Scopus, and Web of Science. The initial search was conducted on December 19, 2024 and the results were subsequently updated with a final search performed on December 19, 2025. In addition, the reference list of all included articles and relevant reviews was manually screened in accordance with PRISMA recommendations, in order to ensure completeness of study identification [16]. Mesh keywords and free text words were used in the research process, along with a few Boolean operators (AND, OR). The following protocol was used for PubMed: (“Dental Implants”[MeSH] OR “Dental Implants”[All Fields] OR “dental implant”[All Fields] OR “oral implant”[All Fields]) AND (“Implant Stability Quotient”[All Fields] OR “ISQ”[All Fields] OR “Resonance Frequency Analysis”[MeSH] OR “Resonance Frequency Analysis”[All Fields] OR “RFA”[All Fields]) AND (“Torque”[MeSH] OR “Torque”[All Fields] OR “Insertion Torque”[All Fields] OR “Marginal Bone Loss”[All Fields] OR “Bone Loss”[All Fields] OR “Implant Survival”[All Fields] OR “Implant Success”[All Fields]). Search strategies for each specific database are available in Table S1. The retrieved references were imported into EndNote software, which automatically removed duplicates; the resulting list was then manually screened to identify and eliminate any remaining duplicates.

### 2.2 Eligibility Criteria

No publication year restriction was applied to the search. Studies were screened and included based on the following criteria:

1. **Population:** Human subjects over 18 years of age, of any gender, and systemically healthy, defined as the absence of uncontrolled systemic diseases or conditions known to affect bone metabolism or wound healing (such as uncontrolled diabetes, metabolic bone disorders, immunosuppression, ongoing chemotherapy or radiotherapy, antiresorptive drugs).
2. **Intervention:** Implant‐supported rehabilitation of partial edentulism, regardless of insertion site or loading protocol (immediate, early, or delayed).
3. **Comparisons/Outcomes:**
  ○ Studies comparing IT with ISQ at the time of implant placement.
  ○ Studies comparing ISQ values with MBL, assessed radiographically or clinically at follow‐up.
  ○ Studies evaluating the association between ISQ and implant survival or success rate (with success defined as the absence of mobility, infection, or pathological bone loss).
4. **Study design:** Randomized controlled trials (RCTs), prospective and retrospective cohort studies, case–control studies, and observational studies (comparative studies, single‐arm studies, and case series) that reported statistical analyses assessing the association between ISQ and at least one predefined clinical parameter (IT, MBL, survival, or success).
5. **Sample size:** Minimum of 20 patients per study.

### 2.3 Focused PIO Question and Effect Measure

(P) Population: systemically healthy adult human subjects undergoing implant therapy.

(I) Intervention: quantification of the ISQ values during the course of the intervention/restoration.

(O) Outcome: association with intraoperative and postoperative surrogate parameters and clinical outcomes.

The targeted clinical question of this systematic review was formatted according to the PIO (Population, Intervention, Outcome) framework: “In patients undergoing dental implant placement, are ISQ values associated with intraoperative and postoperative surrogate parameters (IT and MBL) and with clinical outcomes such as survival and success rates?”

### 2.4 Reference Screening and Inclusion

Two authors (AT and VCAC) independently screened the resulting list for eligible references to be included in this systematic review, according to the inclusion/exclusion criteria listed above. In the first instance, only the title and abstract were assessed, and suitable studies were further evaluated through full‐text appraisal. Inter‐reviewer agreement during the full‐text assessment phase was calculated using the kappa coefficient (*κ*) as a measure of concordance [17]. The third author (GT) was involved in making a final decision in case of disagreement.

### 2.5 Data Extraction

Data extraction was carried out independently by two reviewers (AT and VCAC) using items collected through ad hoc extraction sheets. To identify any difference, the two reviewers combined the extraction Excel files in a joint conference with a third reviewer (GT). The third reviewer (GT) acted as referee in case of discordance between the two reviewers. The following information was extracted and recorded in an Excel sheet: author name, year of publication, journal, study design, patient characteristics, arch, sector location, information about implants, surgical technique, regeneration, method for RFA analysis, RFA measurement interval, ISQ values, information about IT, information about MBL, information about survival rate, information about success rate.

### 2.6 Risk of Bias Assessment

Given the heterogeneity of the studies included in the review, different tools were used to assess the risk of bias according to the study design: the Cochrane RoB 2.0 for the RCTs [18]. The ROBINS‐I tool was used for non‐randomized prospective studies, including controlled clinical trials (CCTs) [19]. The Newcastle‐Ottawa Scale (NOS) was applied to studies with retrospective design [20], while the Joanna Briggs Institute (JBI) tool was used for single‐arm studies and case series [21]. Since the scoring and assessment criteria differ between the various tools, the results were normalized to a common qualitative scale (low, moderate, and high risk of bias) to ensure consistency across the different types of studies.

### 2.7 Eligibility Criteria for Quantitative Synthesis

For continuous outcomes (IT and MBL) studies were considered eligible for quantitative synthesis when they reported a correlation coefficient describing the association between ISQ at baseline and at least one of the predefined outcomes. When multiple correlation estimates were reported (e.g., subgroup analyses), only the overall correlation referring to the entire study population was considered for quantitative synthesis. If an overall estimate was not available, the study was not included in the quantitative pooling. Studies were required to provide sufficient statistical information to allow the transformation of correlation coefficients into Fisher's *Z* values and the calculation of the corresponding variance. For studies reporting a statistical analyses between ISQ and implant survival/success, only those providing mean ISQ values with corresponding standard deviations separately for failed and surviving implants were considered eligible for quantitative synthesis. In the absence of adequate data, studies were included in the qualitative synthesis but not in the quantitative pooling.

### 2.8 Statistical Analysis

When correlations were reported as Spearman's coefficients, they were converted to Pearson's coefficients using the formula *r* = 2sin(*ρπ*/6) as described in literature by Rupinski and Dunald [22]. The resulting values were adjusted according to Poom and Wåhlberg [23]. All correlation coefficients were subsequently transformed using Fisher's *Z* transformation prior to pooling. Meta‐analyses were conducted on the Fisher's *Z* scale, and pooled estimates were subsequently back‐transformed to Pearson's *r* for clinical interpretability. To account for potential unit‐of‐analysis errors in studies where multiple implants per patient were reported without appropriate clustering correction, a variance inflation factor (VIF) was applied where necessary, assuming a conservative ICC of 0.05 [24]. Quantitative analysis was conducted using R software, employing the meta and metafor packages. Heterogeneity across studies was assessed using Cochran's Q test and quantified with the *I* ^2^ statistic. A random‐effect model was applied to estimate the pooled effect size. Pooled results were expressed as the mean correlation coefficient, with corresponding 95% confidence intervals, and graphically displayed through a forest plot. Statistical significance was set at *p* < 0.05 for all analyses. Subgroup analyses were performed for correlation‐based meta‐analyses according to sector location, regeneration procedures, surgical stage, and loading protocol. A sensitivity analysis was performed using a leave‐one‐out approach, whereby each study was sequentially excluded to evaluate the influence of individual studies on the pooled effect size and the robustness of the overall findings. Publication bias was assessed through visual inspection of funnel plots and supplemented with the trim‐and‐fill method [25] and Egger's test [26] when appropriate.

For implant survival and implant success, when sufficient data were available, a mean difference (MD) in ISQ between failed and surviving implants was calculated and pooled using a random‐effects model. Heterogeneity was assessed using Cochran's Q test and quantified with the *I* ^2^ statistic. A leave‐one‐out sensitivity analysis was conducted to evaluate the influence of individual studies on the pooled estimate.

The certainty of the evidence for outcomes included in the quantitative synthesis (ISQ‐IT and ISQ‐Survival) was assessed using the Grading of Recommendations Assessment, Development and Evaluation (GRADE) approach [27]. According to GRADE methodology, the body of evidence was evaluated across five domains: risk of bias, inconsistency, indirectness, imprecision, and publication bias.

## 3 Results

### 3.1 Study Selection

**FIGURE 1:**
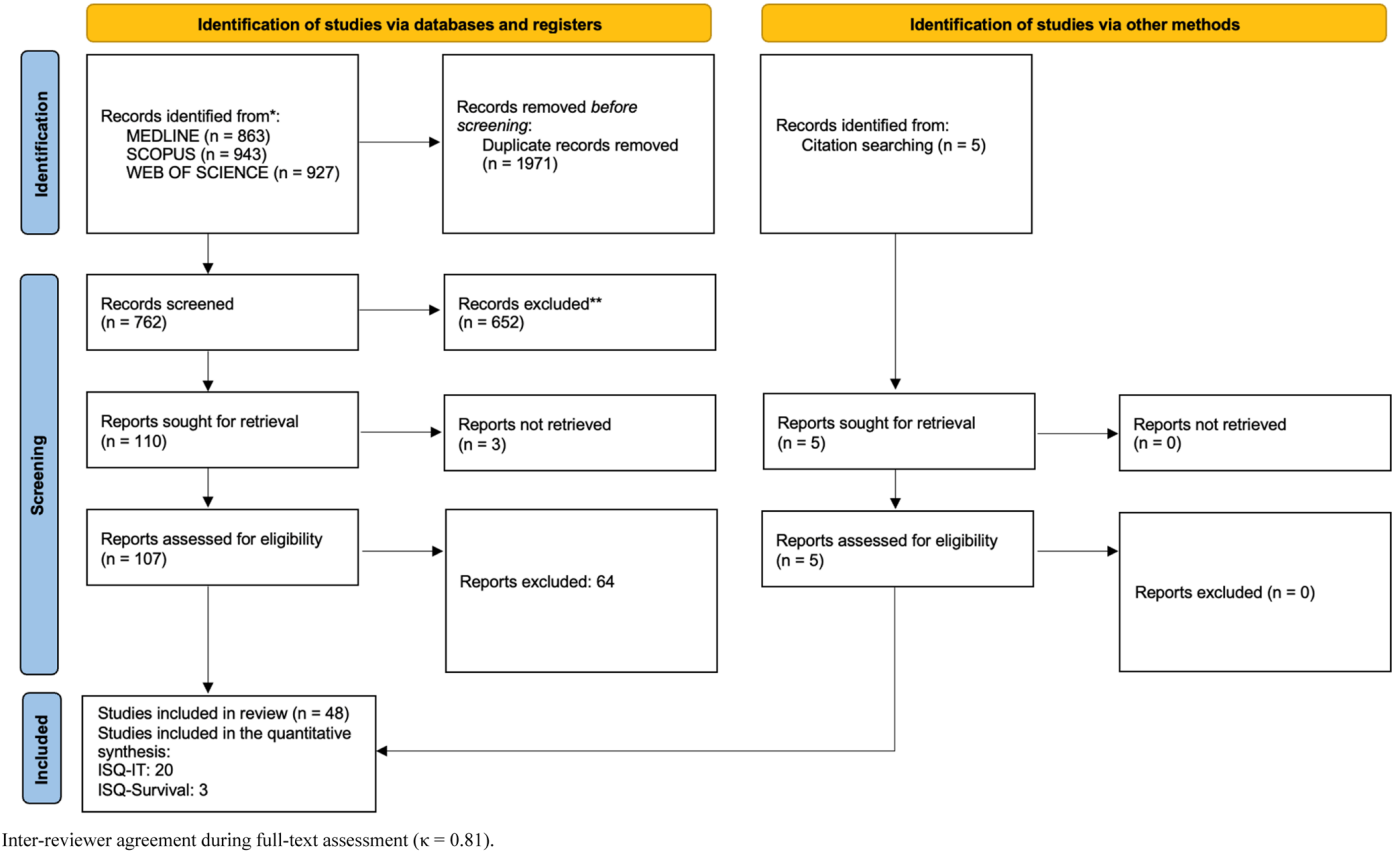
PRISMA 2020 flow diagram of study selection. Inter‐reviewer agreement during full‐text assessment (*κ* = 0.81). *Source:* Page MJ, et al. *BMJ* 2021; 372:N71. doi: 10.1136/bmj.n71.

The online search in the mentioned databases yielded a total of 2733 results (MEDLINE = 863, Scopus = 943, Web of Science = 927). These references were integrated into the EndNote reference software tool. Once duplicates were removed (1971), the titles and abstracts of a total of 762 references were examined and 652 were excluded. One hundred and ten references were evaluated in the full text; however, three full‐text articles were not available. Therefore, 107 full‐text articles were assessed for eligibility, and 64 were excluded (the list and rationale for exclusion are summarized in Table S2). The inter‐reviewer agreement during the full‐text eligibility assessment showed a high level of agreement (*κ* = 0.81). In the end, 43 studies were considered suitable for inclusion in the present systematic review. Additionally, five studies identified through manual search were included, resulting in a final total of 48 studies [5, 6, 13, 15, 17, 28, 29, 30, 31, 32, 33, 34, 35, 36, 37, 38, 39, 40, 41, 42, 43, 44, 45, 46, 47, 48, 49, 50, 51, 52, 53, 54, 55, 56, 57, 58, 59, 60, 61, 62, 63, 64, 65, 66, 67, 68, 69, 70]. Of the 48 studies included in the qualitative synthesis, 20 [5, 13, 31, 33, 35, 38, 40, 42, 44, 45, 50, 54, 56, 57, 60, 61, 63, 65, 67, 70] provided sufficient data to be included in the primary meta‐analysis of ISQ and IT after adjustment for clustering using the VIF. One additional study [62] was included in a sensitivity analysis without VIF correction. Furthermore, three studies provided data for the quantitative analysis of the association between ISQ and implant survival. Figure 1 illustrates the flowchart of the selection process.

### 3.2 Study Characteristics

The 48 included studies were conducted between 2007 and 2025 and exhibit considerable variability in terms of study design and population characteristics. Overall, the studies included a total of 3631 participants, with a mean age ranging between 50 and 60 years in most cases, reported either as an average value [5, 6, 13, 15, 28, 30, 32, 33, 34, 35, 36, 38, 39, 41, 42, 43, 44, 45, 47, 49, 50, 51, 53, 55, 59, 60, 61, 62, 64, 67, 68, 69, 70, 71] or as a range [31, 37, 40, 48, 56, 57, 58, 63]. Gender distribution was also variable: some studies show a higher prevalence of females, while others present a balanced distribution between males and females. Most studies included implant placement in both the maxilla and the mandible [5, 13, 28, 30, 32, 33, 35, 36, 37, 38, 40, 41, 42, 44, 45, 46, 48, 49, 53, 54, 55, 56, 57, 58, 59, 60, 62, 63, 64, 66, 68, 71]. Only nine studies focused exclusively on the maxilla [15, 17, 43, 47, 50, 61, 67, 69, 70], while just three involved only the mandible [6, 29, 65]. Five studies did not report this type of information [31, 34, 39, 51, 54], while the posterior region was the most treated anatomical site [5, 6, 15, 17, 29, 30, 33, 35, 37, 38, 39, 40, 41, 44, 45, 49, 53, 56, 57, 58, 59, 60, 61, 62, 63, 65, 66, 67, 69, 70].

Eleven studies reported performing bone regeneration procedures [15, 35, 43, 53, 54, 55, 56, 59, 61, 64, 70], while four did not provide any data on this aspect [6, 46, 66, 69]. Detailed information on each included study is presented in Table 1. The 48 studies included a total of over 8000 implants placed, with marked heterogeneity in implant brand. Implant diameters ranged from 3 to over 6 mm, with values between 3.75 and 5 mm being the most prevalent. Implant lengths ranged from 7 to 19 mm, with a preference for lengths between 10 and 13 mm. Most studies described the use of a one‐stage surgical approach [5, 6, 15, 29, 30, 33, 34, 35, 42, 45, 47, 48, 49, 51, 53, 54, 55, 58, 63, 65, 67, 68, 71]. Some adopted a two‐stage technique [13, 28, 32, 36, 38, 41, 56, 62], while others included both approaches [17, 43, 61, 64, 70]. Delayed loading was the most frequently used protocol; however, several studies also explored immediate or early loading [5, 17, 29, 35, 38, 39, 47, 51, 53, 54, 56, 58, 63, 68, 70] (Table S3).

**TABLE 1:** Main characteristics of the included studies.

Author name, year	Journal	Study design	No. of patients	Sex	Mean age (Y)	Insert region	Sector location	Regeneration
Rabel et al., 2007 [62]	Clinical Oral Investigations	Prospective Cohort Study	263	98 M—165 W	55.8	Maxilla Mandible	Anterior Posterior	No
Turkyilmaz et al., 2008 [69]	Clinical Implant Dentistry and Related Research	CCT	22	12 M—10 W	49	Maxilla	Posterior	NR
Turkyilmaz et al., 2008 [5]	BMC Oral Health	Retrospective Observational Study	111	56 M—55 W	55 ± 11	Maxilla Mandible	Anterior Posterior	No
Fischer et al., 2009 [47]	Clinical Implant Dentistry and Related Research	Prospective Cohort Study	32	14 M—18 W	54 (Single tooth loss) 65 (two or more missing teeth)	Maxilla	NR	No
Degidi et al., 2010 [57]	The International Journal of Oral & Maxillofacial Implants	Cross‐sectional Study	152	70 M—82 W	Range: 23–83	Maxilla Mandible	Anterior Posterior	No
Karabuda et al., 2011 [51]	Clinical Oral Implants Research	RCT	22	7 M—15 W	46.68	NR	NR	No
Barewal et al., 2012 [63]	International Journal of Oral & Maxillofacial Implants	RCT Stratified	40	15 M—25 W	Range: 20–82	Maxilla Mandible	Posterior	No
Degidi et al., 2012 [40]	Clinical Implant Dentistry and Related Research	Retrospective Cohort Study	1045	368 M—677 W	Range: 18–93	Maxilla Mandible	Anterior Posterior	No
Park et al., 2012 [60]	Journal of Oral Rehabilitation	Prospective Cohort Study	41	25 M—16 W	52.7	Maxilla Mandible	Anterior Posterior	No
Dias et al., 2015 [41]	Clinical Oral Implants Research	Prospective Cohort Study	32	13 M—19 W	44.8	Maxilla Mandible	Anterior Posterior	No
Atieh et al., 2014 [29]	Clinical Oral Implants Research	Prospective Observational Study	28	10 M—18 W	NR	Mandible	Posterior	No
Filho et al., 2014 [46]	The Journal of Oral Implantology	Cross‐sectional Study	27	7 M—20 W	NR	Maxilla Mandible	NR	NR
Kim et al., 2015 [17]	Clinical Oral Implants Research	RCT	21	NR	NR	Maxilla	Posterior	No
De Santis et al., 2016 [39]	International Journal of Oral & Maxillofacial Implants	Prospective Cohort Study	62	27 M—35 W	57	NR	Anterior Posterior	No
Levin et al., 2016 [54]	International Journal of Periodontics & Restorative Dentistry	Retrospective Cohort Study	52	NR	NR	Maxilla Mandible	NR	Yes
Malchiodi et al., 2016 [56]	International Journal of Oral & Maxillofacial Implants	RCT	40	24 M—16 W	Range: 35–75	Maxilla Mandible	Posterior	In all cases, any gap between implant and bone was filled with autogenous bone and Bio‐Oss
Norton et al., 2017 [58]	The International Journal of Oral & Maxillofacial Implants	Closed‐Cohort Prospective Study	22	10 M—12 W	Range: 22–79	Maxilla Mandible	Anterior Posterior	No
Simmons et al., 2017 [67]	Journal of Oral Implantology	Pilot RCT	27	NR	62.3 ± 8.4	Maxilla	Posterior	No
Waechter et al., 2017 [6]	Clinical Implant Dentistry and Related Research	RCCT	20	7 M—13 W	50.8 ± 12.5	Mandible	Anterior Posterior	NR
Zita Gomes et al., 2017 [70]	BioMed Research International	CCT	59	23 M—37 W	56.18 ± 11.76	Maxilla	Posterior	Group A: no rigenerated Group B: partially regenerated Group C: totally regenerated
Baldi et al., 2018 [13]	BioMed Research International	Prospective Cohort Study	75	28 M—47 W	61.6 ± 6.8 (low torque) 57.5 ± 11.3 (medium torque) 4.4 ± 13.2 (high torque)	Maxilla Mandible	NR	No
Homma et al., 2018 [49]	International Journal of Implant Dentistry	CCT	27	11 M—16 W	54.6 ± 12.2	Maxilla Mandible	Posterior	No
Rosen et al., 2018 [64]	International Journal of Implant Dentistry	Retrospective Consecutive Case Series	75	27 M—48 W	61.0 ± 12.5	Maxilla Mandible	Anterior Posterior	Partially
Sarfaraz et al., 2018 [65]	The Journal of Indian Prosthodontic Society	Prospective Observational Study	37	NR	NR	Mandible	Posterior	No
Chen et al., 2019 [37]	International Journal of Prosthodontics	Retrospective Cohort Study	173	65 M—108 W	Range: 21–85	Maxilla Mandible	Anterior Posterior	No
Park et al., 2019 [61]	Implant Dentistry	Prospective Cohort Study	24	12 M—12 W	60.25 ± 9.88	Maxilla	Posterior	Yes
Ab Rahman et al., 2019 [66]	Journal of Contemporary Dental Practice	Prospective Observational Study	21	NR	NR	Maxilla Mandible	Posterior	NR
Dragonas et al., 2020 [43]	Medicina Oral, Patologia Oral, Cirugia Bucal	Retrospective Cohort Study	31	13 M—22 W	61.92 ± 7.45	Maxilla	NR	Partially
Badenes‐Catalán and Pallarés‐Sabater, 2021 [31]	Journal of Oral Implantology	Prospective Cohort Study	114	45 M—69 W	Range: 18–75	NR	NR	No
Bergamo et al., 2021 [33]	Clinical Implant Dentistry and Related Research	CCT	56	26 M—30 W	54.2 ± 3.5	Maxilla Mandible	Anterior Posterior	No
Brouwers et al., 2021 [15]	Clinical Implant Dentistry and Related Research	Prospective Pilot Cohort Study	28	19 M—9 W	60.6	Maxilla	Posterior	Yes
Do Vale Souza et al., 2021 [42]	European Journal of Dentistry	Prospective Observational Study	25	8 M—17 W	50 ± 9	Maxilla Mandible	Anterior	No
Pardo‐Zamora et al., 2021 [59]	International Journal of Environmental Research and Public Health	Prospective Cohort Study	74	35 M—39 W	47.98	Maxilla Mandible	Anterior Posterior	Partially
Cassetta et al., 2022 [36]	International Journal of Oral & Maxillofacial Implants	Prospective Parallel Cohort Study	142	57 M—85 W	55.08 ± 12.43	Maxilla Mandible	NR	No
da Rocha Ferreira et al., 2022 [38]	International Journal of Oral & Maxillofacial Implants	Prospective Cohort Study	51	20 M—31 W	58.53 ± 10.38	Maxilla Mandible	Anterior Posterior	No
Noaman and Bede, 2022 [28]	Journal of Baghdad College of Dentistry	Prospective Observational Study	24	10 M—14 W	47.9 ± 13.64	Maxilla Mandible	NR	No
Feng et al., 2023 [44]	Clinical Oral Implants Research	Cross‐sectional	22	11 M—11 W	57.77 ± 12.83	Maxilla Mandible	Posterior	No
Gehrke et al., 2023 [48]	Medicina	RCT	70	32 M—38 W	Range: 23–68	Maxilla Mandible	NR	No
Bannwart et al., 2024 [32]	Brazilian Oral Research	RCT	20	8 M—12 W	45.54 (External hexagon) 47.38 (Morse Taper)	Maxilla Mandible	NR	No
Canullo et al., 2024 [34]	Clinical Oral Investigations	CCT	36	NR	61.63 ± 9.61 (Control Group) 59.06 ± 11.44 (Test Group)	NR	NR	No
Dkheel et al., 2024 [50]	Journal of Baghdad College of Dentistry	Prospective Observational Study	20	7 M—13 W	39.24 ± 10.42	Maxilla	Anterior	No
Back et al., 2025 [30]	Journal of Clinical Medicine	Retrospective Clinical Study	95	55 M—49 W	57.4 ± 12.3	Maxilla Mandible	Posterior	No
Carosi et al., 2025 [35]	International Journal of Oral Implantology	Prospective Observational Study	31	12 M—19 W	53.81 ± 9.48	Maxilla Mandible	Posterior	Partially
de Moraes Ferreira et al., 2025 [45]	Journal of Dentistry	Randomize Clinical Trial	31	NR	54.1 ± 8.6	Maxilla Mandible	Posterior	No
Lombardi et al., 2025 [55]	Clinical Implant Dentistry and Related Research	Retrospective Clinical Study	73	35 M—38 W	59.3 ± 11.2	Maxilla Mandible	Anterior Posterior	Partially
Kim et al., 2025 [71]	Materials	Prospective clinical study	73	42 M—31 W	58.6 ± 9.4	Maxilla Mandible	NR	No
Ko et al., 2025 [53]	Journal of Periodontal & Implant Science	Retrospective case series	28	15 M—13 W	Within‐ARP: 54.25 ± 11.72; Beyond‐ARP: 52.11 ± 9.63	Maxilla Mandible	Posterior	Yes
Tan et al., 2025 [68]	Journal of Stomatology, Oral and Maxillofacial Surgery	Retrospective Observational study	36	14 M—22 W	50 ± 14.5	Maxilla Mandible	NR	No

Abbreviations: ARP, Alveolar Ridge Preservation; CCT, Controlled Clinical Trial; NR, Not reported; RCT, Randomize Clinical Trial.

### 3.3 ISQ Values and Measurement Time

The major part of studies employed Osstell devices for RFA quantification; just two of them used Penguin RFA [15, 38], while in several cases the specific method was not reported [43, 47, 54, 58]. These data refer to the entire set of studies included in the systematic review. However, only a subset of these was included in the meta‐analysis; among them, just one used Penguin [38]. ISQ values were measured at different time points depending on the study, typically including the time of implant placement, various postoperative intervals (ranging from 1 to 12 months), and later stages such as prosthetic loading or long‐term follow‐up (up to 10 years). Initial ISQ values generally ranged between 60 and 75. In many studies, a progressive increase in ISQ values was observed postoperatively [41, 65]. However, some studies reported a plateau or a slight temporary decrease [34, 36], around 30–45 days after surgery. The measurements from each study are presented in Table S4.

### 3.4 Correlation/Association Between ISQ and IT

A total of 40 studies reported on correlation/association between ISQ and IT [5, 6, 13, 15, 28, 30, 31, 32, 33, 34, 35, 36, 38, 39, 40, 42, 44, 45, 46, 48, 49, 50, 51, 53, 54, 55, 56, 57, 58, 60, 61, 62, 63, 64, 65, 67, 68, 69, 70, 71]. Detailed data on IT, ISQ measurement at baseline (T0), and corresponding statistical analyses are summarized in Table 2. Among these, most studies performed a statistical analysis of the association between IT and ISQ using Pearson's correlation test [6, 15, 31, 32, 33, 35, 36, 42, 44, 46, 48, 50, 53, 54, 56, 60, 61, 62, 65, 67] and Spearman's correlation test [5, 13, 30, 38, 40, 45, 57, 58, 63, 69, 70]. Whereas other studies applied alternative statistical approaches. The reported correlation coefficients ranged from very weak (*r* = 0.002) [44] to very strong (*r* = 0.87) [69]. In the included studies, T0 generally referred to the time of implant placement, whereas T1, T3, and subsequent codes indicated postoperative follow‐up time points (days or months after surgery), as defined by each study protocol. At baseline (T0), most studies reported a positive association between IT and ISQ, although with variable strength. However, when the correlation was assessed at subsequent time points (T1–T3) [15, 28, 34, 36, 39, 42, 44, 48, 54, 56, 65, 70] the association generally weakened, with several studies reporting lower correlation coefficients (*r* < 0.5) and predominantly non‐significant *p* values. Full details regarding the analytical methods, correlation coefficients, statistical significance, and other classification criteria are reported in Tables S5 and S6.

**TABLE 2:** Studies evaluating the relationship between IT and ISQ at T0.

Author name, year	Insertion torque value (Ncm)	ISQ values	Statistical analysis	Correlation coefficient		*p*
Rabel et al., 2007 [62]	30.8 ‡	66.5	Pearson correlation	0.305		NS
Turkyilmaz et al., 2008 [69]	Group C1: 29.7 ± 8	Group C1: 59.5 ± 5	Spearman correlation	0.87		< 0.001
	Group T1: 35.9 ± 6	Group T1: 64.4 ± 3		0.81		< 0.05
	Group: T2: 37.2 ± 7	Group: T2: 66.4 ± 2		0.78		< 0.05
	Group C2: 30.9 ± 7	Group C2: 62.2 ± 5		0.81		< 0.05
	Group T3: 38.5 ± 7	Group T3: 68.3 ± 4		0.84		< 0.05
	Group T4: 41.1 ± 6	Group T4: 70.2 ± 3		0.80		< 0.05
Turkyilmaz et al., 2008 [5]	36.1 ± 8	65.7 ± 9	Spearman correlation	0.764		< 0.001
Degidi et al., 2010 [57]	39.9 ± 20.7 ¶	73.5 ± 10.2	Spearman correlation	0.247		NR
Karabuda et al., 2011 [51]	SLA: 25.49 ± 7.48	SLA: 55.46 ± 8.29	NR	NR		> 0.05
	modSLA: 23.75 ± 7.33	modSLA: 56.63 ± 8.19				
Barewal et al., 2012 [63]	D1/D2: 32.3 ± 11.0 ‡	D1/D2: 72 ± 3.1	Spearman correlation	0.4973		0.0063
	D3: 16.6 ± 7.8 ‡	D3: 70 ± 4.2				
	D4: 10.0 ± 4.6	D4: 58 ± 5.5				
Degidi et al., 2012 [40]	34.82 ± 19.36	71.57 + 10.63	Spearman correlation	0.218		0.0001
Park et al., 2012 [60]	31.17 ‡	71.29	Pearson correlation	0.427		< 0.01
Filho et al., 2014 [46]	D1/D2: 46.27 ± 18.51	D1/D2: 70.09 ± 7.50	Pearson correlation	0.35		0.0213
	D3/D4: 33.62 ± 14.74	D3/D4: 63.66 ± 8.00		0.37		0.0224
De Santis et al., 2016 [39]	76.1 ± 20.8	80.4 ± 8.4	Simple linear regression	0.713		< 0.05
Levin et al., 2016 [54]	28	68	Pearson correlation	0.06		NS
Malchiodi et al., 2016 [56]	Test Group: 46 ± 9.95	Test Group: 61.90 ± 0.99	Pearson correlation	0.83		NR
	Control Group: 52 ± 9.23	Control Group: 66.0 ± 8.25		0.39 0.66 (§§)		
Norton et al., 2017 [58]	12.08 §	MD: 67 ± 11.5	Spearman correlation	MD: 0.281		> 0.05
		BO: 65 ± 11.9		BO: 0.291		> 0.05
Simmons et al., 2017 [67]	NR	Group A: 75.28 ± 7.25	Pearson correlation	0.0509		NR
		Group B: 67.90 ± 8.43				
		Group C: 74.94 ± 5.12				
Waechter et al., 2017 [6]	Tapered: 59.7 ± 14.0	Tapered: 67.86 ± 12.28	Pearson correlation	NR		NR
	Cylindrical: 54.3 ± 13.8	Cylindrical: 62.62 ± 16.99		0.55		0.0108
Zita Gomes et al., 2017 [70]	37.62	69.06	Spearman correlation	0.76		2.2 × 10^−16^
Baldi et al., 2018 [13]	Low torque: 18.8 ± 6.0	Low torque: 71.8 ± 6.6	Spearman correlation	0.342		0.0001
	Medium torque: 41.2 ± 7.2	Medium torque: 75.6 ± 9.2		0.481		
	High torque: 68.2 ± 12.1	High torque: 78.0 ± 6.4		0.121 0.461(§§)		
Homma et al., 2018 [49]	32.7 ± 9.2	67.4 ± 13.61§	Group‐based comparison (*t*‐test/ANOVA)	NR		NS
Rosen et al., 2018 [64]	30.1 ± 7.4	73.6 ± 8.1	Cox proportional hazards regression	NR		> 0.05
Sarfaraz et al., 2018 [65]	39.08 ± 8.688	78.26 ± 5.825	Pearson correlation	0.399		0.000
Park et al., 2019 [61]	37.33 ± 11.09	68.40 ± 11.14 (T0)	Pearson correlation	0.415		0.001
Badenes‐Catalán and Pallarés‐Sabater, 2021 [31]	45.52	74.14	Pearson correlation	0.435		< 0.001
Bergamo et al., 2021 [33]	Osseodensification: 60 ± 3.4	Osseodensification: 73 ± 2.0	Pearson correlation	0.58		< 0.001
	Subtractive Drilling: 35 ± 3.4	Subtractive Drilling: 62 ± 2.0				
Brouwers et al., 2021 [15]	43.6	Osstell (BO): 76.8 ± 5.8	Pearson correlation	0.409		0.015
		Osstell (MD): 78.2 ± 5.5		0.483		0.004
		Penguin (BO): 75.8 ± 5.5		0.416		0.013
		Penguin (MD): 77.5 ± 5.2		0.496		0.003
da Rocha Ferreira et al., 2022 [38]	45 †	78 †	Spearman correlation	0.237		0.0055
Do Vale Souza et al., 2021 [42]	D1: 36.50 ± 3.37 D3: 31.20 ± 5.92	48.24 ± 19.28	Pearson correlation	0.457		0.022
Cassetta et al., 2022 [36]	Group A: 33.63 ††	Group A: 75.04 ††	Pearson correlation	0.494		0.000
	Group B: 34.20 ††	Group B: 72.59 ††				
Noaman and Bede, 2022 [28]	35 > 35	79.03 ± 4.71 §	Group‐based comparison (unpaired *t*‐test)	NR		0.2785
Feng et al., 2023 [44]	31.44 ± 6.54	73.34 ± 7.39	Pearson correlation	0.002		0.988
Gehrke et al., 2023 [48]	DuoCone Maxilla.: 56.21 ± 6.27	DC Maxilla: 77.24 ± 4.55	Pearson correlation	BO: 0.32	MD: 0.35	NR
	DuoCone Mandible: 56.72 ± 6.7	DC Mandible: 78.72 ± 4.84		−0.07	−0.03	
	Maestro Maxilla: 25.30 ± 8.58	MAE Maxilla: 70.52 ± 4.58		0.24	0.27	
	Maestro Mandible: 33.62 ± 8.97	MAE Mandible: 73.48 ± 4.23		−0.12	−0.10	
Bannwart et al., 2024 [32]	Range: 30–44	External hexagon: 76.98 ± 8.12 §	Pearson correlation	0.461		0.031
	≥ 45	Morse Taper: 71.15 ± 11.42 §		0.439		0.041
Canullo et al., 2024 [34]	NINA Group: 32.19 ± 11.70 ‡	NINA Group: 74.57 ± 7.85	Simple linear regression and mixed effects models	NR		> 0.05
	NEO Group: 33.07 ± 9.22 ‡	NEO Group: 77.12 ± 5.83				
Dkheel et al., 2024 [50]	28 ± 11.74	63 ± 18.66	Pearson correlation	0.5		0.027
Back et al., 2025 [30]	Osstem: 36.5 ± 9.4	Osstem: 72.4 ± 5.8	Spearman correlation	0.349		< 0.01
	Toplan: 34.8 ± 8.7	Toplan: 70.1 ± 6.4		0.026		> 0.05
Carosi et al., 2025 [35]	38.7 ± 14.7	71.8 ± 9.3	Pearson correlation	0.661		< 0.001
de Moraes Ferreira et al., 2025 [45]	35 (†)	71.6 ± 6.9	Spearman correlation	0.115		0.442
Kim et al., 2025 [71]	35–44	78.97 ± 5.52	One‐way ANOVA	NR		< 0.05
	45–59					
	≥ 60					
Ko et al., 2025 [53]	Within‐ARP: 17.08 ± 11.1	Within‐ARP: 76.44 ± 6.62 (‡‡)	Pearson correlation	0.415		0.023
	Beyond‐ARP: 33.33 ± 13.39	Beyond‐ARP: 80.66 ± 6.27 (‡‡)				
Lombardi et al., 2025 [55]	34.7 ± 9.1	< 60	Logistic regression	NR		NR (significant)
		≤ 60				
Tan et al., 2025 [68]	< 35	BL: 77.51 ± 5.90	Group‐based comparison (*t*‐test, ANOVA)	NR		< 0.05
	≥ 35	MD: 78.20 ± 6.40				

*Note:* §, Estimate value; §§, Overall coefficient; ¶, The value was calculated on 483 implants; †, This value is a median; ††, The value was calculated on 121 implants (IT < 50); ‡, Peak IT; ‡‡, ISQ measured at 10 weeks post‐implant placement. Group A: Sandblasted and acid‐etched (SAB) surface implants; Group B: Hydrophilic‐modified surface implants; Group C: Nano‐modified surface implants.

Abbreviations: ARP, Alveolar Ridge Preservation; BO, Bucco‐orally; MD, Mesio‐distally; NR, No reported; NS, Not significant.

### 3.5 Correlation/Association Between ISQ and MBL

The relationship between ISQ and MBL was assessed in eight different studies [34, 37, 39, 41, 47, 58, 59, 66]. Considerable heterogeneity was observed in both the timing of ISQ assessment and the statistical approaches adopted. Two of these studies employed the Spearman's test [47, 66], while two used the Pearson's correlation test [41, 59]. Three studies utilized regression models [34, 37, 39], and only one study used non‐parametric tests (Mann–Whitney *U* and Fisher's exact) [58]. Although some research reported correlation coefficients, many did not provide specific numerical values. In these eight studies, T0 generally referred to the time of implant placement, whereas subsequent time codes (e.g., T30) indicated postoperative follow‐up intervals expressed in days or months, according to each study protocol. Among all the combinations analyzed, only the correlation between MBL in the control group and ΔISQ T30‐T0, representing the change in ISQ values between implant placement (T0) and 30 days of healing (T30), in the study by Canullo et al. (2024) reached statistical significance (*p* < 0.05). Full methodological and statistical details are reported in Table 3.

**TABLE 3:** Studies evaluating the relationship between IT and MBL.

Author name, year	Measurement MBL (mm)	T2 – T1 (MBL)	ISQ value	Measurement time ISQ	Statistical analysis	Correlation coefficient	*p*
Fischer et al., 2009 [47]	1.1 ± 1.0 †	T1: implant insertion T2: 1 year post‐surgery	63.3 ± 6.1	Implant insertion 1‐year post‐surgery ΔISQ (baseline‐1 year)	Spearman correlation	NR	> 0.05
			66.8 ± 5.6			NR	> 0.05
			+3.3 ± 5.0			NR	> 0.05
Dias et al., 2015 [41]	−0.47 ± 0.77 (T1‐T3)	T1: uncovering T2: loading T3: 1‐year post‐loading	1.3 ± 7.22	ΔISQ (1‐year post‐loading—uncovering)	Pearson correlation	0.18	0.109
De Santis et al., 2016 [39]	0.7 ± 0.5	T1: prosthetic loading T2: 36 months post‐loading	80.4 ± 8.4	Implant insertion	Multiple linear regression	NR	> 0.05
Norton et al., 2017 [58]	Mesial: 0.06 ‡ Distal: 0.09 ‡	T1: implant insertion T2: 1 year post‐surgery	Mesiodistally: 67 ± 11.5 Buccolingually: 65 ± 11.9	Implant insertion	Mann–Whitney *U* test and Fisher	NR	> 0.05
Chen et al., 2019 [37]	0.22 ± 0.516	T1: implant insertion T2: 1 year post‐surgery	66.1 ± 7.95	1 year	Mixed‐model analysis	NR	0.211
Ab Rahman et al., 2019 [66]	Mesial: 0.786 Distal: 0.8	T1: prosthetic loading T2: 6 months post‐loading	66.2 ± 2.561	6 months post‐loading	Spearman correlation	Mesial: 0.047 Distal: 0.025	0.836 0.914
Pardo‐Zamora et al., 2021 [59]	Short implants: −0.263 ± 0.244 Standard implants: −0.305 ± 0.272	T1: implant insertion T2: prosthetic loading	Short implants: −0.745 ± 2.192 Standard implants: −0.057 ± 2.796	ΔISQ1 (prosthetic loading—implant insertion)	Pearson correlation	NR	0.324
	Short implants: −0.184 ± 0.191 Standard implants: −0.412 ± 0.588	T1: prosthetic loading T2: 1‐year post‐loading	Short implants: +0.298 ± 1.876 Standard implants: +0.654 ± 1.781	ΔISQ2 (1 year post‐loading—prosthetic loading)	Pearson correlation	NR	NR
Canullo et al., 2024 [34]	Control Group: 0.30 ± 0.49 Test Group: 0.31 ± 0.35	T1: implant insertion T2: 6 months post‐surgery	Control Group: 77.12 ± 5.83 Test Group: 74.57 ± 7.85	Implant insertion	Mixed‐effects linear regression	−0.0079	0.367
			Control Group: −3.78 ± 7.50 Test Group: +0.21 ± 3.97	ΔISQ T30‐T0	Simple regression	Negative Positive	< 0.05 > 0.05
			Control Group: −3.68 ± 8.82 Test Group: +0.40 ± 4.36	ΔISQ T45‐T0	Simple regression	Negative Positive	> 0.05 > 0.05

*Note:* †, The value was calculated on 48 implants; ‡, The value was calculated on 29 implants.

Abbreviations: NR, No reported; T1, 1st measurement; T2, 2nd measurement; T30, 30 days post‐surgery; T45, 45 days post‐surgery.

### 3.6 Association Between ISQ and Survival/Success

Five studies assessed the correlation between ISQ and implant survival [5, 29, 37, 43, 64]. Survival rates were generally high across studies, ranging from approximately 85% to over 97%, with follow‐up periods varying from 3 months to 10 years. Considerable heterogeneity was observed in the statistical approaches adopted, including non‐parametric group comparisons, ROC curve analyses, *t*‐tests, and Cox regression models. Most studies consistently reported lower ISQ values in failed implants compared with surviving ones. Turkyilmaz et al. [5] observed a significant difference between surviving (67.1 ± 7) and failed implants (46.5 ± 4; *p* < 0.001). Similarly, Chen et al. [37], with a 10‐year follow‐up, reported significantly lower ISQ values in failed implants compared with surviving implants (*p* < 0.05). Atieh et al. [29] evaluated the predictive value of ISQ using ROC analysis. While ISQ measured at implant insertion was not significantly associated with survival (*p* = 0.70), ISQ assessed at 8 weeks showed significant predictive capability (*p* = 0.001), suggesting that the timing of measurement may influence prognostic value. In contrast, Dragonas et al. [43] did not observe a statistically significant association between ISQ and survival (*p* = 0.2881). Rosen et al. [64] assessed survival using Cox regression; however, although ISQ values were reported, no extractable correlation coefficients were provided. All detailed information, including the number of failed implants, survival/success rates, follow‐up duration, and statistical methods, is reported in Tables 4 and 5.

**TABLE 4:** Association between ISQ values and implant survival outcomes.

Author name, year	Survival implants/Total implants	Survival rate	Follow‐up	ISQ values of survival implants	ISQ values of failed implants	Statistical analysis	*p*
Turkyilmaz et al., 2008 [5]	280/300	93.3%	3.7 years	67.1 ± 7	46.5 ± 4	Mann–Whitney *U* test	< 0.001
Atieh et al., 2014 [29]	24/28 22/28	85.7% 78.6%	3 months 1 year	79.5	NR	ROC Analysis	Implant insertion = 0.70 8 weeks = 0.001
Rosen et al., 2018 [64]	83/86	97.7% 97.7% 94.8%	1 year 5 years 7 years	73.6 ± 8.2	75.0 ± 5.0	Cox regression	NR
Chen et al., 2019 [37]	362/383	95%	10 years	63.0 ± 10.74	52.3 ± 7.03	Group comparison (failed vs. surviving implants) ROC analysis	< 0.05
Dragonas et al., 2020 [43]	46/48	95.8%	22.8 ± 9.9 months	NR	NR	*t*‐test	0.2881

Abbreviation: NR, No reported.

**TABLE 5:** Association between ISQ values and implant success outcomes.

Author name, year	Success implants/Total implants	Rate	Follow‐up	ISQ values of failed implants	Statistical analysis	Correlation coefficient	*p*
Turkyilmaz, 2008 [5]	280/300	93.3%	Average 3.7 ± 0.7 years	Failed implants: 46.5 ± 4	Mann–Whitney *U* test	NR	< 0.001

Abbreviation: NR, No reported.

### 3.7 Quantitative Analysis

**FIGURE 2:**
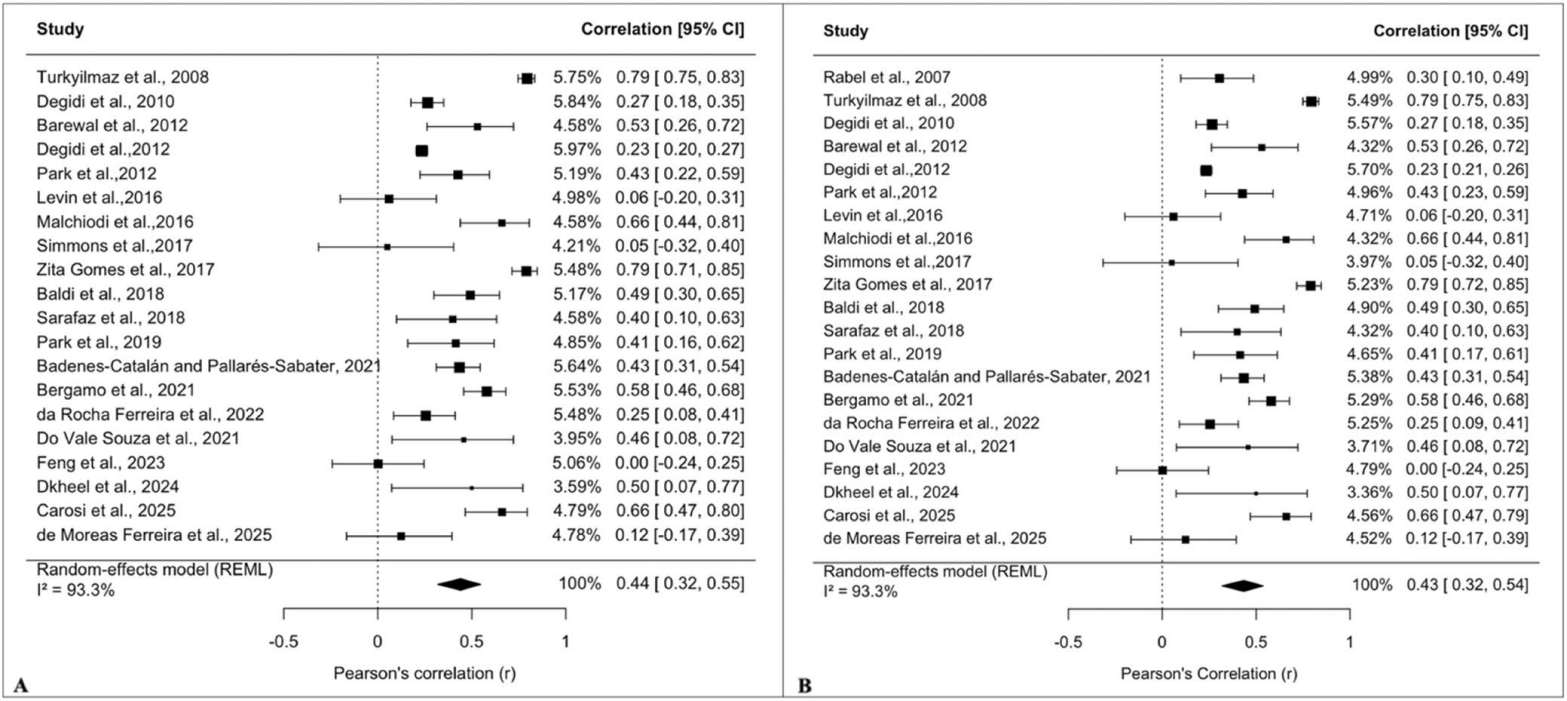
Forest plots of the correlation between ISQ at implant placement and IT. (A) Random‐effects model with VIF correction. (B) Random‐effects model without VIF correction. CI, Confidence interval; ISQ, Implant Stability Quotient; *r*, Correlation coefficient.

The meta‐analysis included 20 [5, 13, 31, 33, 35, 38, 40, 42, 44, 45, 50, 54, 56, 57, 60, 61, 63, 65, 67, 70] studies reporting the overall correlation between ISQ and IT. When necessary, Spearman correlation coefficients were converted into Pearson coefficients using established transformation formulas. All correlation coefficients were then transformed into Fisher's *Z* values prior to pooling and back‐transformed into Pearson's *r* for interpretability. To account for potential clustering effects due to multiple implants placed within the same patient, a VIF correction was applied when appropriate. Using a random‐effects model (REML), the pooled analysis demonstrated a moderate positive correlation between ISQ and IT (*r* = 0.44; 95% CI: 0.32–0.55), which was statistically significant. Heterogeneity among studies was high (*I* ^2^ = 93.3%), indicating substantial variability across the included studies (Figure 2A). To explore the influence of the VIF adjustment, an additional meta‐analysis was conducted without applying VIF correction. In this model, one additional eligible study (Rabel et al., 2007 [62]), which lacked the necessary data for VIF computation, was included. The pooled correlation remained consistent (*r* = 0.43; 95% CI: 0.32–0.54; *I* ^2^ = 93.3%), confirming the stability of the effect estimate (Figure 2B). To further assess the robustness of the primary findings, a leave‐one‐out sensitivity analysis was conducted on the VIF‐adjusted model. Sequential exclusion of individual studies resulted in pooled correlation coefficients ranging from *r* = 0.403 to *r* = 0.456, with a maximum variation (Δ*r*) of ±0.03 compared to the overall estimate. Importantly, the direction and statistical significance of the association remained unchanged across all iterations. Although heterogeneity slightly decreased in some models (*I* ^2^ range: 81.8%–87.8%), substantial variability persisted, indicating that the high heterogeneity was not driven by any single study. Detailed results of the leave‐one‐out analysis are reported in Table S7. Given the persistent high heterogeneity, subgroup analyses were performed to explore potential sources of variability. Studies were stratified according to regeneration procedure, implant position (anterior vs. posterior), surgical protocol (one‐stage vs. two‐stage), and loading protocol (early vs. delayed loading). No statistically significant differences were observed between regenerated and non‐regenerated sites (*p* = 0.58), anterior versus posterior regions (*p* = 0.89), or one‐stage versus two‐stage protocols (*p* = 0.52). Across these subgroups, the correlation between ISQ and IT remained consistently positive. In contrast, a statistically significant difference emerged according to loading protocol, with studies adopting early loading demonstrating a stronger pooled correlation (*r* = 0.76) compared with those using delayed loading (*r* = 0.40). Nevertheless, moderate heterogeneity persisted within subgroups. These analyses were based on unbalanced subgroup distributions and should therefore be interpreted with caution. Detailed subgroup results are presented in Table S8. Subgroup analyses for additional clinically relevant variables were not feasible, as most studies reported mixed populations without providing stratified correlation coefficients. In addition to exploring heterogeneity, potential small‐study effects were assessed to determine whether the pooled estimate might have been influenced by publication bias. Publication bias was evaluated through visual inspection of the funnel plot and Egger's regression test. Egger's test did not indicate evidence of small‐study effects (*z* = −0.35, *p* = 0.728). The trim‐and‐fill procedure identified one potentially missing study on the right side of the funnel plot. After adjustment, the pooled correlation slightly increased to *r* = 0.46 (95% CI: 0.34–0.57), compared with the original estimate of *r* = 0.44 (95% CI: 0.32–0.55). The minimal difference between adjusted and unadjusted estimates suggests that potential publication bias did not materially influence the overall findings. Nevertheless, given the substantial heterogeneity observed, interpretation of funnel plot–based methods should be approached with caution.

**FIGURE 3:**
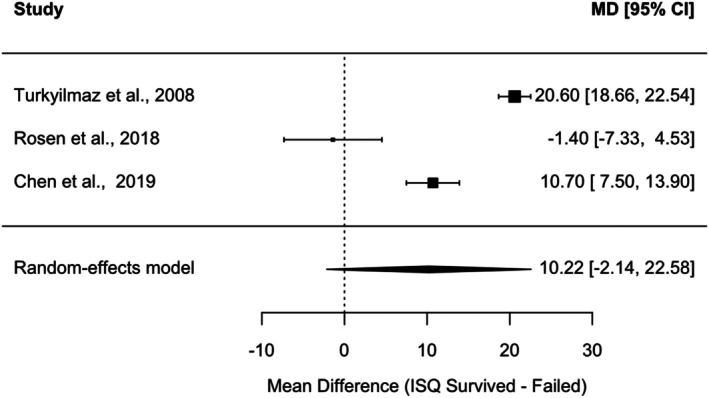
Forest plot of the random‐effects meta‐analysis comparing baseline ISQ values between surviving and failed implants.

Three studies [37, 64, 69] met the predefined eligibility criteria and were included in a random‐effects meta‐analysis comparing baseline ISQ values between surviving and failed implants. The pooled mean difference (MD) in ISQ (survived minus failed implants) was 10.22 (95% CI: −2.14 to 22.58). No statistically significant difference was observed between groups (Figure 3). Substantial heterogeneity was observed across studies. A leave‐one‐out sensitivity analysis demonstrated instability of the pooled estimate. Sequential omission of individual studies yielded mean differences ranging from 4.92 to 15.74, with statistical significance varying depending on the study removed. Heterogeneity remained high across models, indicating considerable between‐study variability. Therefore, these findings should be interpreted with caution.

Although several studies evaluated the relationship between ISQ and MBL, quantitative pooling was not feasible due to the absence of consistently reported correlation coefficients, heterogeneity in follow‐up time points, and variability in statistical analyses. For implant success, only one study [5] met the eligibility criteria for quantitative synthesis; therefore, no meta‐analysis was performed. Findings for both outcomes are presented descriptively.

The certainty of the evidence was evaluated using the GRADE framework (Table S9). For ISQ‐IT, the overall certainty of evidence was rated as very low. The evidence was downgraded due to serious concerns regarding risk of bias and inconsistency, while indirectness and imprecision were not considered serious. For ISQ‐Survival, the certainty of evidence was also rated as very low due to serious inconsistency and imprecision, in addition to the limited number of included studies. No GRADE assessment was performed for MBL and implant success, as quantitative synthesis was not feasible.

### 3.8 Risk of Bias of the Included Studies

**FIGURE 4:**
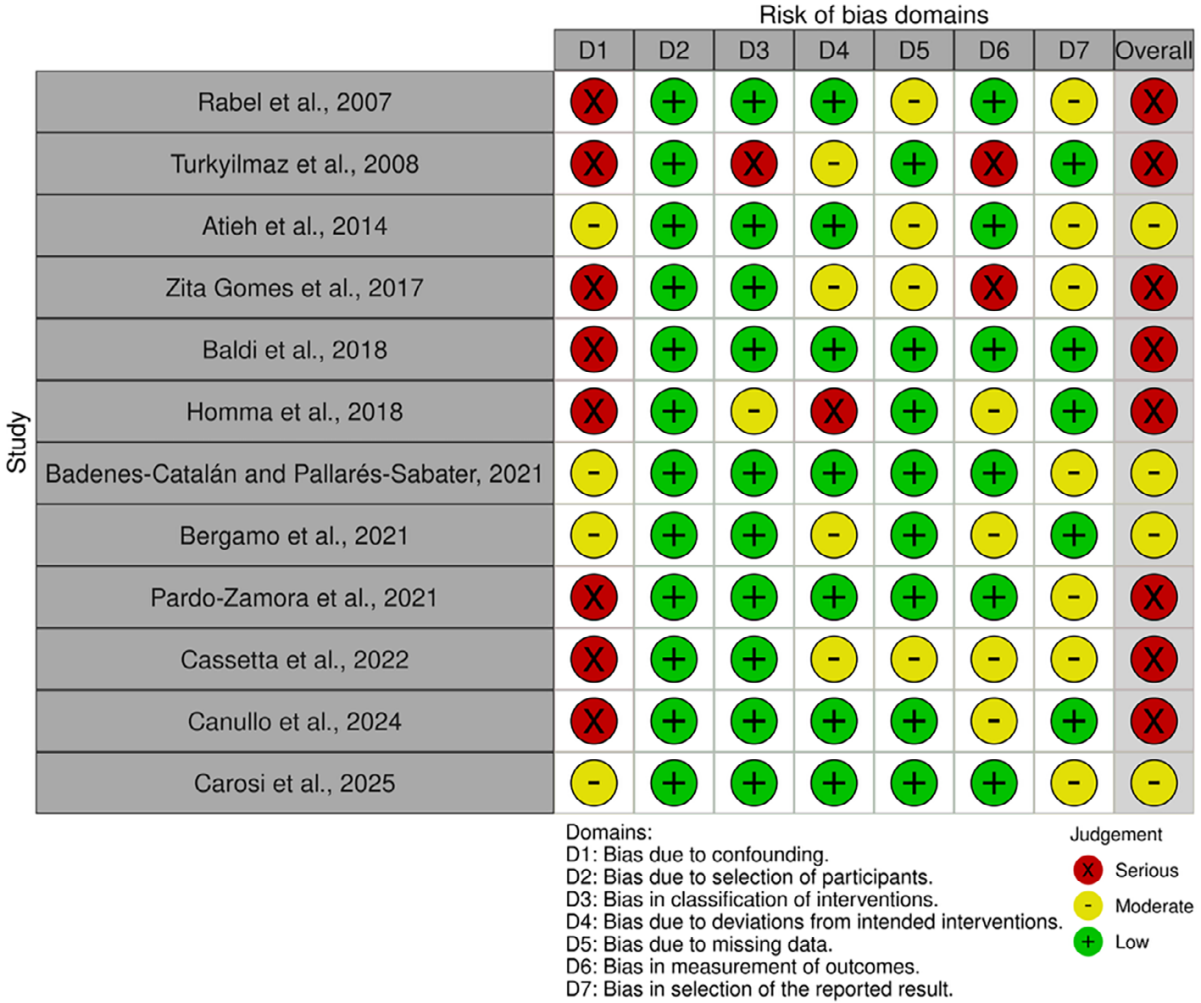
Risk of bias assessment of non‐randomized prospective studies, including CCTs. Plot generated with robvis tool [72].

**FIGURE 5:**
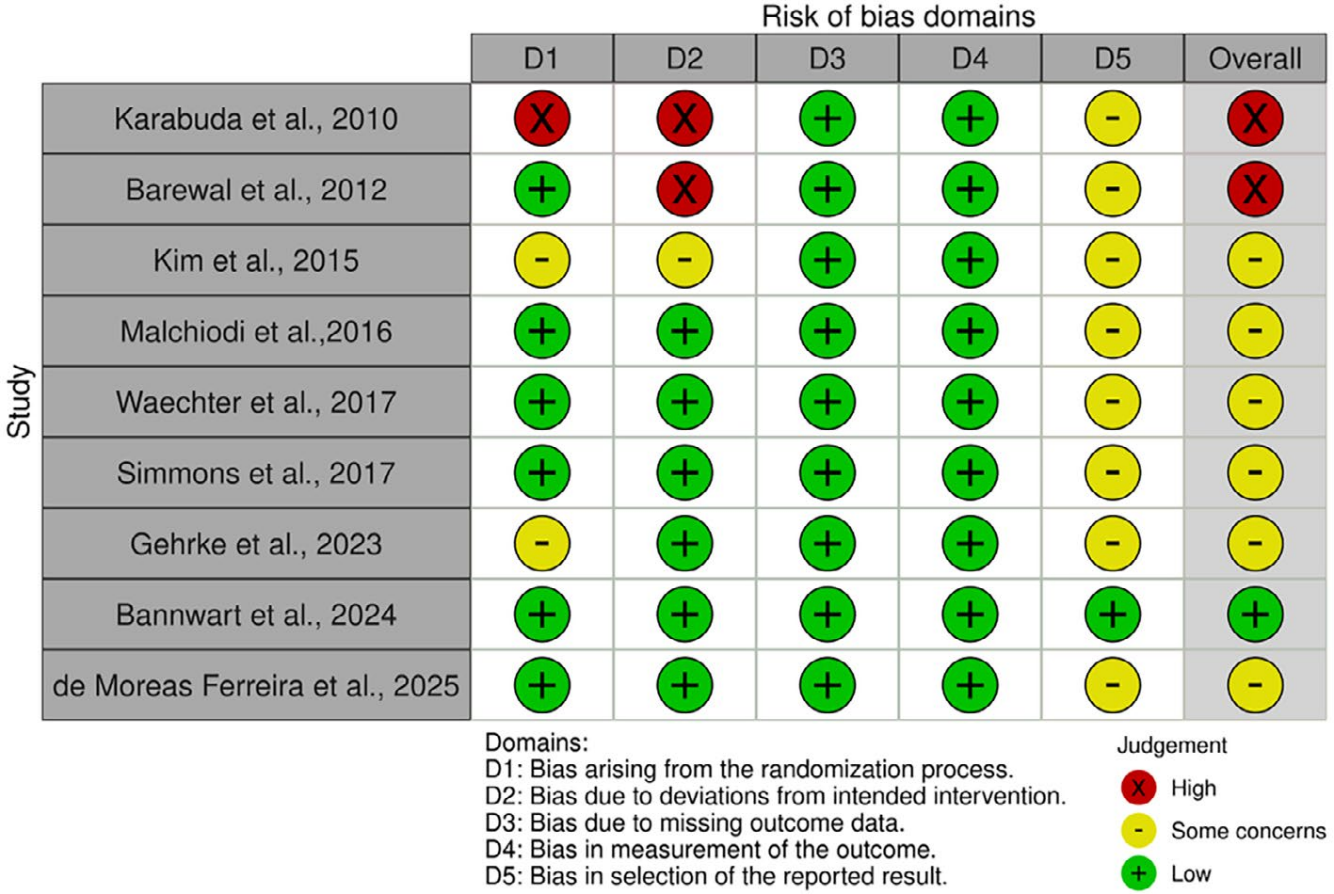
Risk of bias assessment of RCTs included in the review. Plot generated with robvis tool [72].

The risk of bias was assessed using design‐specific tools, and detailed results are reported in Figures 4 and 5 and in Supporting Information (Tables S10 and S11). Non‐randomized prospective studies, including CCTs, were evaluated using the ROBINS‐I tool (Figure 4) [13, 29, 31, 33, 34, 35, 36, 49, 59, 62, 69, 70]. Overall, the risk of bias ranged from moderate to serious. Studies rated at serious risk mainly showed concerns related to bias due to confounding and, in some cases, bias in the measurement of outcomes. Regarding RCTs [6, 17, 32, 45, 48, 51, 56, 63, 67] evaluated with the RoB 2 tool, the overall risk of bias ranged from low to high (Figure 5). Two studies (Karabuda et al. [51] and Barewal et al. [63]) were judged at high risk of bias, mainly due to concerns related to the randomization process and deviations from intended interventions, respectively. Most of the remaining trials were classified as presenting some concerns, while only one study [32] showed an overall low risk of bias. Studies with retrospective design [5, 30, 37, 40, 43, 54, 55, 68, 71], evaluated using the NOS, were predominantly classified as low risk of bias according to the predefined conversion criteria. Single‐arm, cross‐sectional studies [15, 28, 38, 39, 41, 42, 44, 46, 47, 50, 53, 57, 58, 60, 61, 64, 65, 66] assessed with the JBI tool generally demonstrated low to moderate risk of bias.

## 4 Discussion

In recent years, ISQ has gained increasing clinical relevance as a potential decision‐making tool in implant rehabilitation [70]. In this study, we systematically analyzed the available literature to assess whether, and to what extent, the ISQ, measured through RFA, can serve as a reliable diagnostic and prognostic indicator. The meta‐analysis assessing the correlation between ISQ and IT identified a moderate and statistically significant association (*r* = 0.44) between the two parameters, confirming that both contribute to the evaluation of primary implant stability. However, it is essential to distinguish between statistical association and clinical interchangeability. IT measures the rotational frictional resistance encountered during implant advancement and is highly sensitive to surgical technique, such as under‐preparation protocols, as well as to implant macrogeometry [42, 48, 57]. In contrast, ISQ quantifies the axial stiffness and lateral stability of the bone‐implant complex [40, 42, 57]. Findings from other studies, such as those by Sarfaraz et al. [65] and Cassetta et al. [36], further support the idea that ISQ and IT reflect different phenomena and should be considered complementary but not interchangeable tools for assessing implant stability. While IT predominantly represents mechanical primary stability, ISQ appears to be more closely related to the structural and biological characteristics of the implant site [73]. The biological plausibility of ISQ as a measure of stiffness is supported by studies demonstrating its dependence on cortical bone integrity [52] and marginal bone morphology [74]. Almeida et al. [75] further reported that different implant surfaces may present identical initial stability values (both IT and ISQ) while exhibiting distinct biological responses at 28 days. This ability to dynamically monitor implant stability over time represents a substantial advantage over IT, which provides information limited to the moment of insertion. Moreover, the repeatability of ISQ measurements at different time points following implant placement makes it particularly suitable for clinical research and longitudinal monitoring of implant stability, allowing serial assessments without interfering with the healing process.

Despite the moderate correlation observed, the high heterogeneity (*I* ^2^ > 90%) indicates that the strength of the ISQ‐IT association varies considerably across different clinical contexts. Subgroup analyses were conducted to explore potential sources of variability, stratifying studies according to implant site, regenerative procedures, surgical protocol, and loading protocol. No statistically significant differences emerged between regenerated and non‐regenerated sites, anterior versus posterior regions, or one‐stage versus two‐stage approaches, with a positive correlation between ISQ and IT consistently maintained across all categories. A significant difference was observed only with respect to the loading protocol, with a stronger correlation reported in studies adopting early loading compared with delayed loading. However, this finding should be interpreted with caution, as the subgroups were numerically unbalanced and heterogeneity remained moderate even within individual categories. The fact that stratification according to clinically relevant variables did not substantially reduce heterogeneity suggests the presence of effect modifiers not systematically reported in the primary studies, such as differences in implant macrogeometry or actual bone density.

The robustness of the quantitative analysis was supported by the application of the VIF to account for potential intra‐patient clustering in cases involving multiple implants. This adjustment did not materially modify the pooled effect size but ensured a more conservative and methodologically appropriate estimate. Leave‐one‐out analyses confirmed the stability of the aggregated effect, with only minimal variations in the correlation coefficient. Similarly, the trim‐and‐fill method suggested the possible absence of a single study on the right side of the funnel plot. However, adjustment for this hypothetically missing study resulted in only a slight increase in the pooled correlation (from *r* = 0.44 to *r* = 0.46), without substantially altering the overall interpretation of the findings. This suggests that potential small‐study effects or publication bias did not meaningfully influence the pooled estimate. Within the leave‐one‐out framework, exclusion of the only study using a different device (Penguin) [38] did not lead to clinically relevant changes in the ISQ‐IT association, indicating that the overall estimate was not driven by specific measurement system differences. Nevertheless, these findings should be interpreted with caution, given the high heterogeneity among the included studies and the variability of the clinical contexts analyzed. Furthermore, the Pearson correlation coefficient used in the analysis assumes a linear and homogeneous relationship across the entire sample and does not account for potential effect modifiers such as bone density, implant macrogeometry, or loading protocol. In cases where Spearman's coefficients were reported, they were converted into Pearson coefficients using validated formulas; however, this transformation represents an approximation and may have introduced a certain degree of imprecision.

Similarly, the analysis of implant survival revealed a mean difference of approximately 10 ISQ units between surviving and failed implants; however, this difference did not reach statistical significance and showed marked instability in the sensitivity analyses. The lack of statistical significance and the instability of the effect observed in the leave‐one‐out analyses suggest that the current evidence is insufficient to support an independent prognostic role for ISQ. At the same time, these findings highlight the need for studies with standardized reporting and adequate statistical power.

Interpretation of the findings must also take into account the risk of bias of the included studies. The majority of studies were judged to have a moderate or serious risk of bias, primarily due to uncontrolled confounding factors, lack of standardization in surgical protocols, and limited management of intra‐patient clustering. Only a minority of studies were considered to have an overall low risk of bias. This methodological heterogeneity likely contributed to the substantial inconsistency observed in the meta‐analysis and supports the classification of the certainty of evidence as “Very Low” according to the GRADE framework for both quantitative analyses.

Furthermore, the scarcity of data for other outcomes, particularly MBL and implant success, limited the possibility of conducting robust quantitative syntheses.

Building on these considerations, a qualitative assessment of MBL indicates that the available evidence points to a weak or absent correlation with ISQ values. This finding is consistent with the conclusions of Chen et al. [10], who reported that ISQ does not represent a reliable predictor of long‐term MBL. From a biomechanical perspective, several factors may explain why absolute ISQ values do not accurately reflect MBL. First, as suggested by Chen et al. [10], ISQ measures the overall stiffness of the entire bone‐implant complex. MBL often involves only a small portion of the effective implant length engaged in bone; consequently, its impact on the overall resonance frequency may be biomechanically modest compared with the stability provided by the implant body anchored in the basal bone. Second, secondary stability does not depend exclusively on the crestal bone level but rather on the total BIC, as also supported by Mayer et al. [76]. Dias et al. [41] further demonstrated that ISQ values may increase over time as a result of secondary bone maturation, even in the presence of concomitant physiological marginal bone remodeling. This suggests that deeper bone remodeling and increased BIC along the apical portions of the implant may biomechanically compensate for crestal bone loss, thereby maintaining or even increasing the overall stiffness measured by RFA. Although static ISQ values appear to show limited association with MBL, the temporal dynamics of implant stability may provide different insights. The only statistically significant finding among the analyzed studies was reported by Canullo et al. [34], in which the variation in stability between implant placement and 30 days of healing (ΔISQ T30‐T0) in the control group was significantly correlated with MBL at 6 months. This suggests that the prognostic value may lie not in the absolute ISQ value, but rather in the trajectory of stability over time, particularly during the critical phase of the so‐called “stability dip,” which longitudinal evidence indicates typically reaches its minimum around the third postoperative week [77]. A pronounced decrease in stability during the transition from primary to secondary stability may reflect more intense or less efficient bone remodeling, potentially translating into greater MBL after loading. Overall, the inability to perform a quantitative synthesis due to heterogeneity in follow‐up timing and statistical approaches further supports the notion that MBL is a complex, multifactorial phenomenon. As emphasized by Monje et al. [3], biological, inflammatory, and prosthetic loading variables influence the crestal bone in a non‐linear manner that is difficult to attribute to a single biomechanical parameter. ISQ therefore appears to function more appropriately as a longitudinal monitoring tool for bone turnover and interface maturation rather than as a standalone prognostic predictor of MBL.

## 5 Conclusion

Within the limitations of the present systematic review, ISQ shows a moderate and significant correlation with IT, supporting its role as a complementary indicator of primary implant stability. No statistically significant difference in baseline ISQ values between failed and surviving implants was observed. However, substantial heterogeneity and the overall very low certainty of evidence limit the strength of these results. Its predictive value for MBL and implant success remains limited; however, heterogeneity and low certainty of evidence limit clinical interpretability. Overall, current evidence does not support the use of static ISQ values as an independent prognostic predictor of implant outcomes. Rather, ISQ appears to be more appropriately interpreted as a longitudinal monitoring parameter of implant stability.

## Author Contributions

A.T.: conceptualization, data curation, investigation, methodology, writing – original draft, writing – review and editing. F.F.: investigation, visualization, writing – review and editing. V.C.A.C.: data curation, formal analysis, writing – review and editing. K.Z.: methodology, validation, writing – review and editing. M.D.: data curation, visualization, writing – review and editing. G.T.: conceptualization, supervision, validation, writing – review and editing.

## Funding

The authors have nothing to report.

## Ethics Statement

The authors have nothing to report.

## Consent

The authors have nothing to report.

## Conflicts of Interest

The authors declare no conflicts of interest.

## Supporting information


**Table S1:** Search strategy, specific for each screened database.
**Table S2:** References excluded and reason for exclusion.
**Table S3:** Summary of implant‐related characteristics in the included studies.
**Table S4:** ISQ values recorded at different time points across the included studies, along with the RFA device used and measurement intervals.
**Table S5:** Correlation between IT and ISQ at T1. For studies that performed subgroup analyses, correlations are presented separately according to the groups defined by the authors.
**Table S6:** Correlation between IT and ISQ at T2 and T3. For studies that performed subgroup analyses, correlations are presented separately according to the groups defined by the authors.
**Table S7:** Leave‐one‐out sensitivity analysis of the VIF‐adjusted random‐effects model for the correlation between ISQ and insertion torque.
**Table S8:** Results of predefined subgroup analyses performed on the VIF‐adjusted random‐effects model for the correlation between ISQ and insertion torque.
**Table S9:** Summary of Findings (GRADE assessment).
**Table S10:** Risk of bias assessment for studies with a retrospective design (NOS).
**Table S11:** Risk of bias assessment of single‐arm studies and cross‐sectional studies included in the review.

## Data Availability

All data generated or analyzed during this study are included in this published article and its Supporting Information files.
