# Supplementary material for: Resonance Frequency Analysis and Clinical Outcomes in Implant Dentistry: A Systematic Review and Meta‐Analysis
Source: Clin Implant Dent Relat Res. 2026 May 12;28:e70156. doi: 10.1111/cid.70156 (PMC13163645; doi:10.1111/cid.70156)
Supplement: Supplementary file 1 — Table S1: Search strategy, specific for each screened database. Table S2: References excluded and reason for exclusion. Table S3: Summary of implant‐related characteristics in the included studies. Table S4: ISQ values recorded at different time points across the included studies, along with the RFA device used and measurement intervals. Table S5: Correlation between IT and ISQ at T1. For studies that performed subgroup analyses, correlations are presented separately according to the groups defined by the authors. Table S6: Correlation between IT and ISQ at T2 and T3. For studies that performed subgroup analyses, correlations are presented separately according to the groups defined by the authors. Table S7: Leave‐one‐out sensitivity analysis of the VIF‐adjusted random‐effects model for the correlation between ISQ and insertion torque. Table S8: Results of predefined subgroup analyses performed on the VIF‐adjusted random‐effects model for the correlation between ISQ and insertion torque. Table S9: Summary of Findings (GRADE assessment). Table S10: Risk of bias assessment for studies with a retrospective design (NOS). Table S11: Risk of bias assessment of single‐arm studies and cross‐sectional studies included in the review. [file CID-28-0-s001.docx]

**Supplemental Table S1** Search strategy, specific for each screened database.

| **Search strategy** |
| --- |
|  |
| **PubMed** |
| ("Dental Implants"[MeSH] OR "Dental Implants"[All Fields] OR "dental implant"[All Fields] OR "oral implant"[All Fields]) AND ("Implant Stability Quotient"[All Fields] OR "ISQ"[All Fields] OR "Resonance Frequency Analysis"[MeSH] OR "Resonance Frequency Analysis"[All Fields] OR "RFA"[All Fields]) AND ("Torque"[MeSH] OR "Torque"[All Fields] OR "Insertion Torque"[All Fields] OR "Marginal Bone Loss"[All Fields] OR "Bone Loss"[All Fields] OR "Implant Survival"[All Fields] OR "Implant Success"[All Fields]) |
|  |
| **Scopus** |
| TITLE-ABS-KEY ( ( "Dental Implants" OR "dental implant" OR "oral implant" ) AND ( "Implant Stability Quotient" OR isq OR "Resonance Frequency Analysis" OR rfa ) AND ( torque OR "Insertion Torque" OR "Marginal Bone Loss" OR "Bone Loss" OR "Implant Survival" OR "Implant Success" ) ) |
|  |
| **Web of Science** |
| TS=("Dental Implants" OR "dental implant" OR "oral implant") AND  TS=("Implant Stability Quotient" OR ISQ OR "Resonance Frequency Analysis" OR RFA) AND  TS=(Torque OR "Insertion Torque" OR "Marginal Bone Loss" OR "Bone Loss" OR "Implant Survival" OR "Implant Success") |

**Supplemental Table S2** References excluded and reason for exclusion.

| **Author name, Year** | **Journal** | **Title** | **Reason For Exclusion** |
| --- | --- | --- | --- |
| Turkyilmaz et al., 2006 | Journal of Oral Rehabilitation | Assessment of correlation between computerized tomography values of the bone, and maximum torque and resonance frequency values at dental implant placement | PZ-NC |
| Al-Nawas et al., 2007 | Clinical Implant Dentistry and Related Research | Turned, Machined Versus Double-Etched Dental Implants In Vivo | No comparison between variables |
| Shiigai et al., 2007 | Journal of Oral Implantology | Pilot study in the identification of stability values for determining immediate and early loading of implants | No comparison between variables |
| Turkyilmaz et al., 2007 | Journal of Clinical Periodontology | Relations between the bone density values from computerized tomography, and implant stability parameters: a clinical study of 230 regular platform implants | PZ-NC |
| Yamaguchi et al., 2008 | Quintessence International | Resonance frequency analysis of long-term implant success in the posterior partially edentulous mandible | No comparison between variables |
| Bornstein et al., 2009 | Clinical Implant Dentistry and Related Research | Early Loading of Nonsubmerged Titanium Implants with a Chemically Modified Sand-Blasted and Acid-Etched Surface: 6-Month Results of a Prospective Case Series Study in the Posterior Mandible Focusing on Peri-Implant Crestal Bone Changes and Implant Stability Quotient (ISQ) Values | No comparison between variables |
| Bogaerde et al., 2010 | Clinical Implant Dentistry and Related Research | Immediate/Early Function of Neoss Implants Placed in Maxillas and Posterior Mandibles: An 18-Month Prospective Case Series Study | No comparison between variables |
| Rodrigo et al., 2010 | Clinical Oral Implants Research | Diagnosis of implant stability and its impact on implant survival: a prospective case series study | Lack of data |
| Winter et al., 2010 | The International Journal of Oral & Maxillofacial Implants | Parameters of Implant Stability Measurements Based on Resonance Frequency and Damping Capacity: A Comparative Finite Element Analysis | Doesn't meet the inclusion criteria |
| Alghamdi et al., 2011 | Journal of Oral and Maxillofacial Surgery | Undersized Implant Site Preparation to Enhance Primary Implant Stability in Poor Bone Density: A Prospective Clinical Study | No comparison between variables |
| Ho et al., 2011 | Clinical Oral Implants Research | Clinical and radiographic evaluation of Nobel Active TM dental implants | No comparison between variables |
| Blaszczyszyn et al., 2012 | Biomed Tech | Immediate loading of an implant with fine threaded neck - bone resorption and clinical outcome of single tooth restorations in the maxilla | No comparison between variables |
| Huber et al., 2012 | Clinical Implant Dentistry and Related Research | A 1-Year Controlled Clinical Trial of Immediate Implants Placed in Fresh Extraction Sockets: Stability Measurements and Crestal Bone Level Changes | No comparison between variables |
| Atieh et al., 2013 | Clinical Oral Implants Research | Immediate single implant restorations in mandibular molar extraction sockets: a controlled clinical trial | No comparison between variables |
| Becker et al., 2013 | Clinical Implant Dentistry and Related Research | Prospective Clinical Trial Evaluating a New Implant System for Implant Survival, Implant Stability and Radiographic Bone Change | No comparison between variables |
| Nienkemper et al., 2013 | Head and Face Medicine | Impact of mini-implant length on stability at the initial healing period: a controlled clinical study | PZ-NC |
| Herekar et al., 2014 | Journal of Prosthetic Dentistry | A correlation between bone (B), insertion torque (IT), and implant stability (S): BITS Score | No comparison between variables |
| Mantena et al., 2014 | Journal of Dental Research | Evaluation of crestal bone loss and stability of immediate functional loading versus immediate non-functional loading of single-mandibular posterior implants: A pilot randomized controlled clinical trial | No comparison between variables |
| Kim et al., 2014 | International Journal of Periodontics & Restorative Dentistry | Prospective Observational Study of the Early Loading of Nanostructured Calcium Phosphate-Coated Tapered Implants in the Mandible and Maxilla | No comparison between variables |
| Tatli et al., 2014 | Journal of Craniofacial Surger | Does Cone Beam Computed TomographyYDerived Bone Density Give Predictable Data About Stability Changes of Immediately Loaded Implants?: A 1-Year Resonance Frequency Follow-up Study | PZ-NC |
| Krafft et al., 2015 | Journal of Oral Implantology | Osstell resonance frequency measurement values as a prognostic factor in implant dentistry | No comparison between variables |
| Huang et al., 2016 | Therapeutics and Clinical Risk Management | Mathematical evaluation of the influence of multiple factors on implant stability quotient values in clinical practice: a retrospective study | Lack of data |
| De Val et al., 2017 | Journal of Craniofacial Surgery | Effects of Surface Treatment Modification and Implant Design in Implants Placed Crestal and Subcrestally Applying Delayed Loading Protocol | No comparison between variables |
| Gehrke et al., 2017 | Clinical Implant Dentistry and Related Research | Stability and Crestal Bone Behavior Following Simultaneous Placement of Multiple Dental Implants (Two or More) with the Bone Splitting Technique: A Clinical and Radiographic Evaluation | No comparison between variables |
| Hadzik et al., 2017 | Annals of Anatomy | Comparative evaluation of the effectiveness of the implantation in the lateral part of the mandible between short tissue level (TE) and bone level (BL) implant systems | PZ-NC |
| Huang et al., 2017 | PLOS ONE | Multivariate linear regression analysis to identify general factors for quantitative predictions of implant stability quotient values | Lack of data |
| Rossi et al., 2017 | Clinical Oral Implants Research | 6-mm-long implants loaded with fiber- reinforced composite resin-bonded fixed prostheses (FRCRBFDPs) - A 5-year prospective study | No comparison between variables |
| de Oliveira Nicolau Mantovani et al., 2018 | Oral and Maxillofacial Surgery | Influence of cortical bone anchorage on the primary stability of dental implants | PZ-NC |
| Göthberg et al., 2018 | Clinical Implant Dentistry and Related Research | Bone and soft tissue outcomes, risk factors, and complications of implant-supported prostheses: 5-Years RCT with different abutment types and loading protocols | Lack of data |
| Rosen et al., 2018 | International Journal of Implant Dentistry | 1-7 year retrospective follow-up on consecutively placed 7-mm-long dental implants with an electrowetted surface | PZ-NC |
| Silva et al., 2018 | Journal of Oral Implantology | Assessment of dental implants stability in areas previously submitted to maxillary sinus elevation | PZ-NC |
| Andersson et al., 2019 | International Journal of Dentistry | Factors Influencing Resonance Frequency Analysis (RFA) Measurements and 5-Year Survival of Neoss Dental Implants | PZ-NC |
| Bavetta et al., 2019 | BioMed Research International | A Retrospective Study on Insertion Torque and Implant Stability Quotient (ISQ) as Stability Parameters for Immediate Loading of Implants in Fresh Extraction Sockets | PZ-NC |
| Díaz-Sánchez Rosa-María et al., 2019 | Medicina Oral Patología Oral y Cirurgía Bucal | Analysis of marginal bone loss and implant stability quotient by resonance frequency analysis in different osteointegrated implant systems. Randomized prospective clinical trial | PZ-NC |
| Zumstein et al., 2019 | Clinical Implant Dentistry and Related Research | Factors influencing marginal bone loss at a hydrophilic implant design placed with or without GBR procedures: A 5-year retrospective study | PZ-NC |
| Bianconi et al., 2020 | International Journal of Oral Implantology | Bone modifications around porous trabecular implants inserted with or without primary stability 2 months after tooth extraction: A 3-year controlled trial | No comparison between variables |
| Corvino et al., 2020 | International Journal of Oral Implantology | Clinical and radiological outcomes of implants with two different connection configurations: A randomised controlled trial | No comparison between variables |
| Díaz-Castro et al., 2020 | International Journal of Environmental Research and Public Health | A Non-Interventional Study Documenting Use and Success of Tissue Level Implants | No comparison between variables |
| Lee et al., 2020 | Materials | Early Loading of Mandibular Molar Single Implants: 1 Year Results of a Randomized Controlled Clinical Trial | No comparison between variables |
| Takechi et al., 2020 | Materials | Morphological Evaluation of Bone by CT to Determine Primary Stability-Clinical Study | Doesn't meet the inclusion criteria |
| Sreerama et al., 2021 | Journal of Pharmacy and Bioallied Sciences | Assessment of the Effect of Bone Density on Implant Stability: A Clinical Study | Lack of data |
| Steiner et al., 2021 | Quintessence International | Observational study on primary and secondary stability of two current bone level implant designs | No comparison between variables |
| Antequera-Diaz et al., 2022 | Clinical Oral Investigations | Intra‐ and inter‐operator concordance of the resonance frequency analysis. A cross‐sectional and prospective clinical study | PZ-NC |
| Oliveira et al., 2022 | International Journal of Oral and Maxillofacial Surgery | The correlation of different methods for the assessment of bone quality in vivo: an observational study | Lack of data |
| Tian et al., 2022 | Oral Surgery, Oral Medicine, Oral Pathology, and Oral Radiology | Correlation between bone volume fraction in posterior implant area and initial implant stability | No comparison between variables |
| Alassah et al., 2023 | Journal of the Mechanical Behavior of Biomedical Materials | Evaluation of the implant stability and the marginal bone level changes during the first three months of dental implant healing process: A prospective clinical study | No comparison between variables |
| Marchio et al., 2023 | International Journal of Periodontics & Restorative Dentistry | Resonance Frequency Analysis to Assess the Stability of Immediate Implants: A Retrospective Clinical Trial | PZ-NC |
| Alzoubi et al., 2024 | Heliyon | The relationship between the secondary implant stability quotient and oxidized implant-related factors: A retrospective study | PZ-NC |
| Alzoubi et al., 2024 | Journal of Oral Implantology | Implant Stability After Graftless Motor-Driven Crestal Sinus Elevation: A Cohort Study | Full text not available |
| Barbosa et al., 2024 | Brazilian Dental Journal | Effect of different thread configurations on hydrophilic implant stability. A split-mouth RCT | No comparison between variables |
| Jamil et al., 2024 | Evidence-Based Dentistry | Unlocking implant success: the impact of surgical techniques on primary stability in the posterior maxilla | Inappropriate type of publication |
| Rathi et al., 2024 | Indian Journal of Dental Research | Effect of Densah Burs on Primary and Secondary Stability of Immediately Loaded Implants in Addition to Crestal Bone Loss and Gingival Probing Depth - An Evaluative Study | No comparison between variables |
| Ruppin et al., 2024 | International Journal of Implant Dentistry | High insertion torque versus regular insertion torque: early crestal bone changes on dental implants in relation to primary stability - a retrospective clinical study | PZ-NC |
| Shahood et al., 2024 | BMC Oral Health | CGF with Bio-Oss collagen as grafting materials for simultaneous implant placement after osteome sinus floor elevation: a prospective study | No comparison between variables |
| Ahmed Galal Sadek et al., 2025 | BMC Oral Health | Peri-implant bone behavior after single drilling technique versus undersized drilling technique of immediately loaded implant in posterior maxilla: a one-year prospective study | No comparison between variables |
| Calciolari et al., 2025 | Clinical Oral Implants Research | Radiographic Peri-Implant Bone Changes in OsteoporoticWomen Treated With a Ti-Zr, Bone Level Tapered Implant With a Hydrophilic Surface: A 12-Month Prospective Case-Series | No comparison between variables |
| Chakraborty et al., 2025 | Bioinformation | The impact of alveolar bone density and width on primary implant stability | No comparison between variables |
| Charoenniwassakul et al., 2025 | Journal of Clinical Periodontology | Effect of Dental Implant Design on Stability During EarlyHealing: A Randomised Controlled Trial | No comparison between variables |
| Do Vale Sauza et al., 2025 | Journal of Long-Term Effects of Medical Implants | Influence of Torque Type and Bone Loss on the Stability Quotient of Two Implants with Prostheses | Full text not available |
| Dounis et al., 2025 | Clinical and Experimental Dental Research | Dental Implants Placed in Fresh Human Extraction Sockets Without Osteotomy: A Case Series | No comparison between variables |
| Gong et al., 2025 | International Dental Journal | Impact of Age-Related Bone Density Variations on Dental Implant Stability and Success Rates: A Retrospective Analysis | No comparison between variables |
| Gokila Vani et al., 2025 | The Journal of Oral Implantology | Horizontal Augmentation via Ridge Splitting and Expansion for Implant Placement in Atrophic Sites: A Prospective Non-Randomized Controlled Trial | Full text not available |
| Hakobyan et al., 2025 | Bulletin of Stomatology and Maxillofacial Surgery | Evaluation of the effectiveness of immediate dental implants placed in extraxtion sockets with periapical pathology. A controlled case series | No comparison between variables |
| Mangalekar et al., 2025 | Bioinformation | Analyzing stability parameters for assessing immediate and early loading of implants | No comparison between variables |
| Martín-Martín et al., 2025 | Journal of Clinical Medicine | Influence of Healing Abutment Height on Secondary Implant Stability Using Resonance Frequency Analysis: A Prospective Clinical Study | No comparison between variables |
| Porta-Ferrer et al., 2025 | Clinical Oral Implants Research | Influence of Neck Design on Peri-Implant Hard and Soft Tissues: A 12-Month Randomized Clinical Trial of Immediately Placed Implants | No comparison between variables |
| Vabanaboyina et al., 2025 | Cureus | COVID-19 as a Risk Factor for Peri-Implant Disease: A Prospective Clinical Study | No comparison between variables |

(PZ-NC) Patient criteria not compatible

**Supplemental Table S3** Summary of implant-related characteristics in the included studies.

| **Author Name, Year** | **N° OF IMPLANTS** | **IMPLANT BRAND** | **DIAMETER OF THE IMPLANT (mm)** | **LENGHTH OF THE IMPLANT (mm)** | **ONE STAGE/  TWO STAGE** | **LOAD** |
| --- | --- | --- | --- | --- | --- | --- |
| Rabel et al., 2007 | 602 | Dentsply Sirona, Camlog | 3.5 3.8 4.3 4.5 5 5.5 6 | 8 9 9.5 11 13 14 16 17 | Two stage | Delayed loading |
| Turkyilmaz et al., 2008 | 60 | Nobel Biocare | 3.75 4 | 10 11.5 | NR | NR |
| Turkyilmaz et al., 2008 *(BMC)* | 300 | Nobel Biocare | 3.75 4 | 8.5 10 11.5 13 15 | One stage | Early loading |
| Fischer et al., 2009 | 53 | Nobel Biocare | 4.3 5 | 10 13 16 | One stage | Early loading |
| Degidi et al., 2010 | 514 | Dentsply Friadent | 3 3.4 3.8 4.5 5.5 | 8 9.5 11 13 15 18 | NR | NR |
| Karabuda et al., 2010 | 96 | Straumann | 4.1 | From 8 to 12 | One stage | Early loading |
| Barewal et al., 2012 | 40 | Astra Tech | 4 | 11 13 | One stage | Immediate, early and delayed loading |
| Degidi et al., 2012 | 4135 | Dentsply Friadent | 3 3.4 3.8 4.5 5.5 | 8 9.5 11 13 15 18 | NR | NR |
| Park et al., 2012 | 81 | Brånemark implants, ITI implants | < 4 >4 | <11.5 >11.5 | NR | NR |
| Dias et al., 2014 | 78 | Neodent | 3.75 | 9 11 13 15 17 19 | Two stage | Delayed loading |
| Atieh et al., 2014 | 28 | MAX Southern Implants | 8 9 | 7 9 11 | One stage | Immediate loading |
| Filho et al., 2014 | 80 | S.I.N. | NR | NR | NR | NR |
| Kim et al., 2015 | 46 | Straumann | 4.1 4.8 | 10 12 | One stage Two stage | Immediate and delayed loading |
| De Santis et al., 2016 | 144 | Nobel Biocare | 3.5 4.3 5 | 8.5 10 11.5 13 15 | Two stage | Early loading |
| Levin et al., 2016 | 59 | Astra Tech | From 3 to 4.8 | From 11 to 15 | One stage | Immediate and delayed loading |
| Malchiodi et al., 2016 | 40 | Sybron Implant Solution | 3.3 4.1 | 9 11 13 | Two stage | Immediate and delayed loading |
| Norton et al., 2017 | 30 | Astra Tech | 3.6 4.2 4.5 4.8 4 | 9 11 13 15 17 | One stage | Immediate and delayed loading |
| Simmons et al., 2017 | 30 | NR | 4.0 | 8 | One stage | Delayed loading |
| Waechter et al., 2017 | 40 | Signo Vinces | 4 4.60 | 10 | One stage | Delayed loading |
| Zita Gomes et al., 2017 | 133 | Megagen | 3.5 4 4.5 5 5.5 6 | 7 8.5 10 11.5 13 15 | One stage Two stage | Immediate and delayed loading |
| Baldi et al., 2018 | 75 | Megagen | 4 | 10 | Two stage | Delayed loading |
| Homma et al., 2018 | 33 | GC Dental | 3.8 4.4 | 8 10 12 | One stage | NR |
| Rosen et al., 2018 | 86 | NR | 3.5 4.0 4.5 5.0 5.5 6.0 | 7.0 | One stage Two stage | Delayed loading |
| Sarfaraz et al., 2018 | 40 | Nobel Biocare | 3.5  4.3  5 | 10  11.5  13 | One stage | Delayed loading |
| Chen et al., 2019 | 393 | NR | From 3.23 to 8 | From 7 to 18 | NR | NR |
| Park et al., 2019 | 55 | Osstem | 4 4.5 5 | 8.5 10 11.5 | One stage Two stage | Delayed loading |
| Rahman et al., 2019 | 21 | NR | NR | NR | NR | Delayed loading |
| Dragonas et al., 2020 | 48 | Zimmer Biomet, Nobel Biocare, Astra Tech, Straumann, Biohorizons | <4 from 4 to 4.9 > 5 | >10 <10 | One stage Two stage | NR |
| Badenes-Catalán and Pallarés-Sabater, 2021 | 194 | Nobel Active, Klockner Essential, Straumann BLT | 3.3 3.5 4 4.1 4.3 4.5 5 | 8 8.5 10 11.5 12 13 15 | NR | Delayed loading |
| Bergamo et al., 2021 | 150 | S.I.N., Zimmer Biomet, NeoBiotech | >3 to <3.75 >3.75 to <5 >5 | >6 to <10 >10 to <13 > 13 | One stage | Delayed loading |
| Brouwers et al., 2021 | 28 | BEGO Implant Systems | 3.75 4.1 4.5 5.5 | 8.5 10 11.5 13 15 | One stage | Delayed loading |
| Do Vale Souza et al., 2021 | 25 | DSP Biomedical | 3.75 | 11.5 | One stage | Delayed loading |
| Pardo-Zamora et al., 2021 | 99 | Avinent Implant System | 3.5 4 4.5 5 | 7 8.5 10 11.5 13 15 | NR | Delayed loading |
| Cassetta et al., 2022 | 268 | SM Implant System, UF II Implant System | 3.8 4.1 4.5 5 5.3 | 10 12 14 | Two stage | Delayed loading |
| da Rocha Ferreira et al., 2022 | 137 | Nobel Biocare | 3.5 3.75 4.3 5 | <10 >10 | NR | Immediate and delayed loading |
| Noaman et al., 2022 | 42 | Dentium | NR | NR | Two stage | Delayed loading |
| Feng et al., 2023 | 65 | Straumann | NR | NR | NR | NR |
| Gehrke et al., 2023 | 100 | Implacil De Bortoli | 3.5 4 | 9 11 13 | One stage | Delayed loading |
| Bannwart et al., 2024 | 44 | DSP Biomedical | 4 | 11.5 | Two stage | Delayed loading |
| Canullo et al., 2024 | 60 | AlphaBio Tec | NR | NR | One stage | Delayed loading |
| Dkheel et al., 2024 | 20 | Binnovation | 3.5 4 | 11  13 | NR | NR |
| Back et al., 2025 | 144 | Osstem Toplan | 3.5  5.0 | 8  12 | One stage | Delayed loading |
| Carosi et al., 2025 | 50 | Straumann | 3.75 4.5 5.5 | 6 12 | One stage | Early loading |
| de Moraes Ferreira et al., 2025 | 48 | Straumann | 3.3  4.8 | 8  12 | One stage | Delayed loading |
| Ko et al., 2025 | 30 | Astra Osseospeed Dentium Superline | >4-<5 >5 | <8 8-10  >10 | One stage | Early loading |
| Kim et al., 2025 | 73 | NR | 4  5 | 8  12 | One stage | Delayed loading |
| Lombardi et al., 2025 | 110 | Nobel Biocare | 3.5  5.0 | 8  13 | One stage | Delayed loading |
| Tan et al., 2025 | 82 | NR | < 4 | < 10 | One stage | Early loading |

NR: not reported

**Supplemental Table S4** ISQ values recorded at different time points across the included studies, along with the RFA device used and measurement intervals.

| **Author Name, Year** | **Method for RFA Analysis** | **ISQ Values** | | | **Measurement Time** |
| --- | --- | --- | --- | --- | --- |
| Rabel et al., 2007 | Osstell | 66.5  66,8 | | | Implant insertion  3 months post-surgery |
| Turkyilmaz et al., 2008 | Osstell | Group C1: 59.5 ± 5  Group T1: 64.4 ± 3 Group T2: 66.4 ± 2 Group C2: 62.2 ± 5 Group T3: 68.3 ± 4 Group T4: 70.2 ± 3 | | | Implant insertion |
| Turkyilmaz et al., 2008 *(BMC)* | Osstell | 65.7 ± 9  66.9 ± 6 §  68.6 ± 7 § | | | Implant insertion  6 months post-surgery  12 months post-surgery |
| Fischer et al., 2009 | NR | 63.3 ± 6.1  64.3 ± 5.3 65.0 ± 4.6 66.8 ± 5.6 | | | Implant insertion  3 months post-surgery  6 months post-surgery  12 months post-surgery |
| Degidi et al., 2010 | Osstell | 73.5 ± 10.2 | | | Implant insertion |
| Karabuda et al., 2010 | Osstell | *SLA*  55.46 ± 8.29  56.08 ± 7.01  55.94 ± 5.95  58.21 ± 5.2  58.47 ± 5.32 | | *modSLA*  56.63 ± 9.19  55.6 ± 9.07  55.4 ± 6.5  58.15 ± 6.52  60.42 ± 6.82 | Implant insertion  1 week post-surgery  3 weeks post-surgery  6 weeks post-surgery  Loading stage |
| Barewal et al., 2012 | Osstell | D1/D2: 72 ± 3.1 D3: 70 ± 4.2 D4: 58 ± 5.5 | | | Implant insertion  Implant insertion Implant insertion |
| Degidi et al., 2012 | Osstell | 71.57 ± 10.63 | | | Implant insertion |
| Park et al., 2012 | Osstell | 71.29 | | | Implant insertion |
| Dias et al., 2014 | Osstell | 73.4 ± 10.3 80.2 ± 6.2 81 ± 3.7 81.5 ± 3.7 | | | Implant insertion  At the time of implant uncovering  At the time of rehabilitation  1 year post-loading |
| Atieh et al., 2014 | Osstell | 79.5 72.49 79.71 ¶ | | | Implant insertion 8 weeks post-surgery 1 year post-surgery |
| Filho et al., 2014 | Osstell | *Group A*  70.09 ± 7.50 | | *Group B*  63.66 ± 8.0 | Implant insertion |
| Kim, 2015 | Osstell | *Delayed loading*  66.2 ± 6.5 | | *Immediate loading*  66.8 ± 7.4 | Implant insertion |
| De Santis et al., 2016 | Osstell | 80.4 ± 8.4  71.2 ± 8.2 | | | Implant insertion  Between 3 and 4 months |
| Levin et al., 2016 | NR | 68  73 | | | Implant insertion  10 weeks post-surgery |
| Malchiodi et al., 2016 | Osstell | 63.95 ± 8.81  67.48 ± 5.95 | | | Implant insertion  Prosthetic load |
| Norton et al., 2017 | NR | *MD*  67.23  80.00 | | *BO*  64.53  79.07 | Implant insertion  12 weeks post-surgery |
| Simmons et al., 2017 | Osstell | Group A: 75.28 ± 7.25 Group B: 67.90 ± 8.43 Group C: 74.94 ± 5.12  Group A: 75.06 ± 8.19 Group B: 77.10 ± 4.84 Group C: 77.56 ± 4.51  Group A: 84.45 ± 4.46 Group B: 82.75 ± 4.31 Group C: 83.61 ± 2.03  Group A: 84.15 ± 4.22 Group B: 82.74 ± 4.03 Group C: 83.61 ± 2.04 | | | Implant insertion  6 weeks (††)  6 months (††)    12 months (††) |
| Waechter et al., 2017 | Osstell | *Tapered implants*  67.86 ± 12.28  53.97 ± 17.86  69.29 ± 13.55 77.58 ± 9.59  71.19 ± 9.67  73.58 ± 8.58  78.61 ± 8.85 | | *Cylindrical implants*  62.62 ± 16.99  59.54 ± 14.55  67.65 ± 15.47  77.96 ± 8.12  71.90 ± 12.84  70.87 ± 7.94  76.62 ± 8.52 | Implant insertion  1-week post-surgery  2 weeks post-surgery  3 weeks post-surgery  6 weeks post-surgery  2 months post-surgery  3 months post-surgery |
| Zita Gomes et al., 2017 | Osstell | 69.06  75.35 ‡  71.8 | | | Implant insertion  When primary stability is achieved  60 days post-surgery |
| Baldi et al., 2018 | Osstell | Low Torque Group: 71.8 ± 6.6 Medium Torque Group: 75.6 ± 9.2 High Torque Group: 78.0 ± 6.4 | | | Implant insertion |
| Homma et al., 2018 | Osstell | 68.0 ± 13.7  71.8 ± 8.3  78.0 ± 5.7 | | | Implant insertion  4 weeks post-surgery  1 year post-surgery |
| Rosen et al., 2018 | Osstell | 73.6 ± 8.1 79.4 ± 4.1 77.3 ± 5.0 74.9 ± 5.6 73.3 ± 4.4 | | | Implant insertion  1-4 weeks  5-8 weeks  9-12 weeks  13-16 weeks |
| Sarfaraz et al., 2018 | Osstell | 78.26 ± 5.825  75.03 ± 6.816  75.95 ± 6.089  78.13 ± 5.719  79.5 ± 4.404 | | | Implant insertion  3 weeks post-surgery  7 weeks post-surgery  11 weeks post-surgery  15 weeks post-surgery |
| Chen et al., 2019 | Osstell | 62.2 ±10.87  62.1 ± 8.35  66.1 ± 7.95  65.3 ± 7,86  65.5 ± 6.90  68.8 ± 7.37 | | | Implant insertion Delivery definitive restoration  1 year post-loading  2 years post-loading  5 years post-loading  10 years post-loading |
| Park et al., 2019 | Osstell | 68.40 ± 11.14 82.24 ± 4.75 | | | Implant insertion 1,5 year post-surgery |
| Rahman et al., 2019 | Osstell | 61.7 ± 1.178  63.3 ± 0.995  66.2 ± 2.561 | | | Before loading with crown  3 months post-loading  6 months post-loading |
| Dragonas et al., 2020 | NR | 73.46 ± 8.06  78.44 ± 6.97 | | | Implant insertion  After a healing period of at least 3 months |
| Badenes-Catalán and Pallarés-Sabater, 2021 | Osstell | 74.15 75.90 | | | Implant insertion 3 months post-surgery |
| Bergamo et al., 2021 | Osstell | Osseodensification: 73 ± 2.0 Subtractive Drilling: 62 ± 2.0 | | | Implant insertion  3 weeks post-surgery |
|  |  | Osseodensification: 70 ± 2.0 Subtractive Drilling: 59 ± 2.0 | | |  |
|  |  | Osseodensification: 74 ± 1.5 Subtractive Drilling: 66 ± 1.5 | | | 6 weeks post-surgery |
| Brouwers et al., 2021 | Osstell  Penguin RFA | *Osstell*  BO:76.8 ± 6.0 MD: 78.2 ± 5.5  BO:78.3 ± 5.8 MD: 79.7 ± 4.7  BO:79.6 ± 6.9 MD: 80.3 ± 6.4  BO:81.6 ± 5.4 MD: 82.9 ± 4.6 | | *Penguin RFA*  BO: 75.8 ± 5.5 MD: 77.5 ± 5.2  BO:77.4 ± 6.0 MD: 79.2 ± 4.3  BO:77.8 ± 7.7 MD: 78.9 ± 7.9  BO:82.2 ± 4.9 MD: 83.0 ± 4.9 | Implant insertion  10 days post-surgery  7 weeks post-surgery  17 weeks post-surgery |
| Do Vale Souza et al., 2021 | Osstell | 48.24 ± 19.28  65.96 ± 8.47 | | | Implant insertion  4 months post-surgery |
| Pardo-Zamora et al., 2021 | Osstell | *Short implants*  −0.745 ± 2.192  0.298 ± 1.876 | | *Standard implants*  -0.057 ± 2.796  0.654 ± 1.81 | ∆ ISQ2-ISQ1  ∆ ISQ3-ISQ3 |
| Cassetta et al., 2022 | Osstell | *Group A* 75.04  78.53  81.66  80.86 | | *Group B*  72.59  76.16  80.10  80.75 | Implant insertion  2 months post-surgery  6 months post-loading  12 months post-loading |
| da Rocha Ferreira et al., 2022 | Penguin RFA | 78 † | | | NR |
| Noaman et al., 2022 | Osstell | *Torque 35*  78.93 ± 5.32  73.55 ± 6.40 | | *Torque >35*  80.30 ± 5.26  75.15 ± 6.32 | Implant insertion  4 months post-surgery |
| Feng et al., 2023 | Osstell | 73.34 ± 7.39 80.32 ± 4.58 | | | Implant insertion 3 months post-surgery |
| Gehrke et al., 2023 | Osstell | *Maxilla* DuoCone: 60.5 ± 3.25 Maestro: 61.4 ± 3.67 | *Mandible* DuoCone: 61.9 ± 4.33 Maestro: 62.4 ± 4.19 | | Implant insertion |
|  |  | DuoCone: 64.7 ± 3.09 Maestro: 66.7 ± 2.51 | DuoCone: 66.5 ± 4.71 Maestro: 68.7 ± 2.33 | | 30 days post-surgery |
|  |  | DuoCone: 68.9 ± 2.88 Maestro: 71.1 ± 2.27 | DuoCone: 71.0 ± 4.65 Maestro: 73.3 ± 2.95 | | 45 days post-surgery |
| Bannwart et al., 2024 | Osstell | *External hexagon* 72.18 ± 9.73  79.22 ± 7.33  81.75 ± 9.22  81.77 ± 4.78 | *Morse taper*  63.92 ± 14.08  72.29 ± 11.08  78.33 ± 5.27  75.76 ± 8.50 | | Implant insertion - Torque (30-44)  Implant insertion - Torque (>45)  Post-osseointegration - Torque (30-44)  Post-osseointegration - Torque (>45) |
| Canullo et al., 2024 | Osstell | *Test Group*  74.57 ± 7.85  74.78 ± 7.31  74.97 ± 6.34 | *Control Group*  77.12 ± 5.83  73.33 ± 6.13  73.44 ± 7.89 | | Implant insertion  30 days post-surgery  45 days post-surgery |
| Dkheel et al., 2024 | Osstell | 63 ± 18.66 | | | Implant insertion |
| Back et al., 2025 | Osstell | Osstem: 72.4 ± 5.0  Toplan: 70.1 ± 6.4 | | Osstem: 77.9 ± 4.6  Toplan: 74.5 ± 5.2 | Implant insertion  Before prosthetic loading |
| Carosi et al., 2025 | Osstell | 71.8 ± 9.3  70.4 ± 7.7  74 ± 7.0  76 ± 6.0  77 ± 5.0  79.1 ± 4.9  79.8 ± 3.9  80.7 ± 3.6  81.0 ± 4.0  81.0 ± 4.0  81.7 ± 3.4  82.1 ± 3.5  82.0 ± 3.0 | | | Implant insertion  1 month post-surgery  2 months post-surgery  3 months post-surgery  4 months post-surgery  5 months post-surgery  6 months post-surgery  7 months post-surgery  8 months post-surgery  9 months post-surgery  10 months post-surgery  11 months post-surgery  12 months post-surgery |
| de Moraes Ferreira et al., 2025 | Osstell | 71.6 ± 6.9  75.8 ± 5.7  77.9 ± 4.8  80.1 ± 4.2 | | | Implant insertion  3 months post-surgery  6 months post-surgery  12 months post-surgery |
| Ko et al., 2025 | Osstell | Within-ARP: 76.44 ± 6.62 Beyond-ARP: 80.66 ± 6.27 | | | 10 weeks post-implant placement |
| Kim et al.,  2025 | Osstell | 78.97 ± 5.52  80.39 ± 5.23 80.78 ± 4.78 81.54 ± 5.28  82.31 ± 4.64 83.74 ± 4.36 | | | Implant insertion  1 week post-surgery  2 weeks post-surgery  3 weeks post-surgery  4 weeks post-surgery  8 weeks post-surgery |
| Lombardi et al.,  2025 | Osstell | < 60  >60 | | | Implant insertion |
| Tan et al., 2025 | Osstell | *BO*  77.51± 5.90  79.38 ± 5.80  77.16 ± 5.90  79.29 ± 5.90 | | *MD*  78.20 ± 6.40  79.80 ± 5.70  77.51 ± 5.50  80.11 ± 5.10 | Implant insertion  1-week post-surgery  4 weeks post-surgery  12 weeks post-surgery |

(†) The reported value corresponds to the median
(‡) The value was calculated on 31 implants
(§) The value was calculated on 280 implants
(¶) The value was calculated on 22 implants
(††) The value was calculated on 28 implants
(BO) bucco-orally
(MD) mesio-distally

(ARP) Alveolar Ridge Preservation
(Group A): Sandblasted and acid-etched (SAB) surface implants
(Group B): Hydrophilic-modified surface implants
(Group C): Nano-modified surface implants

**Supplemental Table S5** Correlation between IT and ISQ at T1. For studies that performed subgroup analyses, correlations are presented separately according to the groups defined by the authors.

| **Author Name, Year** | **Insertion Torque Value (Ncm)** | **ISQ Values** | **Statistical Analysis** | **Correlation coefficient** | | **P-value** |
| --- | --- | --- | --- | --- | --- | --- |
| De Santis et al., 2016 | 76.1 ± 20.8 | 71.2 ± 8.2 | Simple linear regression | 0.276 | | <0.05 |
| Levin et al., 2016 | 28 | 73 | Pearson correlation | 0.06 | | NS |
| Malchiodi et al., 2016 | Test Group: 46 ± 9.95 Control Group: 52 ± 9.23 | Test Group: 68.15 ± 5.97 Control Group: 66.80 ± 6.01 | Pearson correlation | NR | | > 0.05 |
| Zita Gomes et al., 2017 | 37.62 | 71.80 | Spearman correlation | 0.67 | | 2.2 x 10^-16^ |
| Sarfaraz et al., 2018 | 39.08 ± 8.688 | 75.03 ± 6.816 | Pearson correlation | 0.376 | | 0.000 |
| Brouwers et al., 2021 | 43.6 | OSSTELL (BO): 78.3 ± 5.8 †  OSSTELL (MD): 79.7 ± 4.7 †  PENGUIN (BO): 77.4 ± 6.0 †  PENGUIN (MD): 79.2 ± 4.3 † | Pearson correlation | 0.031  0.108  0.119  0.091 | | 0.443 0.311  0.297  0.342 |
| Do Vale Souza et al., 2021 | D1: 36.50 ± 3.37  D3: 31.20 ± 5.92 | 65.96 ± 8.47 | Pearson correlation | NR | | 0.308 |
| Cassetta et al., 2022 | Group A: 33.63 § Group B: 34.20 § | Group A: 78.53 § Group B: 76.16 § | Pearson correlation | 0.172 | | 0.059 |
| Noaman et al., 2022 | 35  >35 | 73.55 ± 6.40  75.15 ± 6.32 | T-test | NR | | 0.4194 |
| Feng et al., 2023 | 31.44 ± 6.54 | 80.32 ± 4.58 | Pearson correlation | 0.238 | | 0.057 |
| Gehrke et al., 2023 | DuoCone Maxilla.: 56.21 ± 6.27 DuoCone Mandible: 56.72 ± 6.7 Maestro Maxilla: 25.30 ± 8.58 Maestro Mandible: 33.62 ± 8.97 | DC Maxilla: 77.24 ± 4.55 DC Mandible: 78.72 ± 4.84 MAE Maxilla: 70.52 ± 4.58 MAE Mandible: 73.48 ± 4.23 | Pearson correlation | BO: 0.34  0.008  0.16  -0.06 | MD: 0.35  -0.07  0.01  -0.22 | NR |
| Canullo et al., 2024 | NINA Group: 32.19 ± 11.70 ‡ NEO Group: 33.07 ± 9.22 ‡ | NINA Group: 74.78 ± 7.31 NEO Group: 73.33 ± 6.13 | Simple linear regression and mixed effects models | NR | | > 0.05 |

(BO) Bucco-orally
(MD) Mesio-distally

(†) The value was calculated on 23 implants
(‡) Peak IT

(§) The value was calculated on 121implants (IT < 50)

**Supplemental Table S6** Correlation between IT and ISQ at T2 and T3. For studies that performed subgroup analyses, correlations are presented separately according to the groups defined by the authors.

| **Author Name, Year** | **Insertion Torque Value (Ncm)** | **ISQ Values (T2)** | **Statistical Analysis** | **Correlation coefficient** | | **P-value** | | **ISQ Values (T3)** | | **Statistical Analysis** | | **Correlation coefficient** | | **P-value** | |  |
| --- | --- | --- | --- | --- | --- | --- | --- | --- | --- | --- | --- | --- | --- | --- | --- | --- |
| Sarfaraz et al., 2018 | 39.08 ± 8.688 | 75.95 ± 6.089 | Pearson correlation | 0.372 | | 0.000 | | 78.13 ± 5.719 | | Pearson correlation | | 0.171 | | 0.053 | |  |
| Brouwers et al., 2021 | 43.6 | Osstell (BO): 79.6 ± 6.9 † Osstell (MD): 80.3 ± 6.4 †  Penguin (BO): 77.8 ± 7.7 ‡ Penguin (MD): 78.9 ± 7.9 ‡ | Pearson correlation | 0.305  0.250  0.169  0.149 | | 0.064  0.108  0.208  0.237 | | Osstell (BO): 81.6 ± 5.4 § Osstell (MD): 82.9 ± 4.6 §  Penguin (BO): 82.2 ± 4.9 ‡ Penguin (MD): 83 ± 4.9 ‡ | | Pearson correlation | | 0.255  0.406  0.371  0.340 | | 0.114  0.024  0.037  0.048 | |  |
| Cassetta et al., 2022 | Group A: 33.63 Group B: 34.20 | Group A: 81.66 Group B: 80.10 | Pearson correlation | -0.021 | | 0.817 | | Group A: 80.86 Group B: 80.75 | | Pearson correlation | | 0.199 | | 0.029 | |  |
| Gehrke et al., 2023 | DuoCone Max.: 56.21 ± 6.27 DuoCone Mand.: 56.72 ± 6.7 Maestro Max.: 25.30 ± 8.58 Maestro Mand.: 33.62 ± 8.97 | DC Max: 78.28 ± 4.01 DC Mand: 79.28 ± 3.85 MAE Max: 71.36 ± 4.35 MAE Mand: 74.20 ± 4.14 | Pearson correlation | BO: 0.34  -0.14  -0.01  -0.22 | MD: 0.36  -0.1  -0.04  -0.08 | | NR | | NR | | NR | | NR | | NR | |
| Canullo et al., 2024 | NINA Group: 32.19 ± 11.70 ¶ NEO Group: 33.07 ± 9.22 ¶ | NINA Group: 74.97 ± 6.34 NEO Group: 73.44 ± 7.89 | Simple linear regression and mixed effects models | NR | | >0.05 | | NR | | NR | | NR | | NR | |  |

(BO) Bucco-orally
(MD) Mesio-distally

(†) The value was calculated on 26 implants
(‡) The value was calculated on 25 implants

(§) The value was calculated on 24 implants

(¶) Peak IT

**Supplemental Table S7** Leave-one-out sensitivity analysis of the VIF-adjusted random-effects model for the correlation between ISQ and insertion torque.

| **Omitted study** | **Pooled r** | **95% CI** | **I² (%)** | **Δr** |
| --- | --- | --- | --- | --- |
| Turkyilmaz et al., 2008 | 0.403 | 0.289-0.506 | 82.2 | -0.032 |
| Degidi et al., 2010 | 0.446 | 0.318-0.557 | 86.4 | +0.009 |
| Barewal et al., 2012 | 0.431 | 0.302-0.544 | 87.6 | -0.005 |
| Degidi et al., 2012 | 0.448 | 0.322-0.558 | 81.8 | +0.012 |
| Park et al., 2012 | 0.436 | 0.308-0.549 | 87.7 | +0.001 |
| Levin et al., 2016 | 0.454 | 0.333-0.560 | 86.3 | +0.018 |
| Malchiodi et al., 2016 | 0.422 | 0.295-0.534 | 87.0 | -0.013 |
| Simmons et al., 2017 | 0.451 | 0.329-0.558 | 86.9 | +0.015 |
| Zita Gomes et al., 2017 | 0.407 | 0.290-0.513 | 84.2 | -0.028 |
| Baldi et al., 2018 | 0.432 | 0.303-0.546 | 87.4 | -0.003 |
| Sarafaz et al., 2018 | 0.435 | 0.309-0.550 | 87.7 | +0.002 |
| Park et al., 2019 | 0.437 | 0.309-0.549 | 87.8 | +0.001 |
| Badenes-Catalán and Pallarés-Sabater, 2021 | 0.436 | 0.306-0.550 | 87.1 | +0.000 |
| Bergamo et al., 2021 | 0.427 | 0.298-0.540 | 87.3 | -0.009 |
| da Rocha Ferreira et al., 2022 | 0.445 | 0.318-0.555 | 87.4 | +0.009 |
| Do Vale Souza et al., 2021 | 0.435 | 0.307-0.547 | 87.8 | -0.001 |
| Feng et al., 2023 | 0.456 | 0.338-0.559 | 85.7 | +0.021 |
| Dkheel et al., 2024 | 0.433 | 0.306-0.545 | 87.3 | -0.003 |
| Carosi et al., 2025 | 0.423 | 0.297-0.535 | 87.2 | -0.012 |
| de Moreas Ferreira et al., 2025 | 0.450 | 0.327-0.558 | 86.9 | +0.014 |

r = pooled Pearson correlation; 95% CI = 95% confidence interval; I² = heterogeneity; Δr = change in pooled correlation after omitting each study.

**Supplemental Table S8** Results of predefined subgroup analyses performed on the VIF-adjusted random-effects model for the correlation between ISQ and insertion torque.

| **Variable** | **Subgroup** | **Studies (n)** | **Pooled r** | **95% CI** | **I^2^** | **p-value** |
| --- | --- | --- | --- | --- | --- | --- |
| Regeneration | Yes | 3 | 0.48362 | -0.070 -0.809 | 91.23694 | 0.5811882 |
|  | No | 16 | 0.3927642 | 0.261 - 0.509 | 85.17479 |  |
|  | | | | | | |
| Implant position | Anterior | 2 | 0.4760257 | 0.201 - 0.681 | 0 | 0.8888993 |
|  | Posterior | 9 | 0.4441092 | 0.222 - 0.622 | 82.12513 |  |
|  | | | | | | |
| Surgical protocol | One-stage | 9 | 0.4488072 | 0.236 - 0.620 | 83.16156 | 0.5209029 |
|  | Two-stage | 3 | 0.5649868 | 0.377 - 0.708 | 36.35756 |  |
|  | | | | | | |
| Loading protocol | Early-loading | 2 | 0.7551383 | 0.614 - 0.849 | 46.09546 | 0.0001562701 |
|  | Delayed-loading | 9 | 0.3953134 | 0.271 - 0.505 | 43.57612 |  |

r = pooled Pearson correlation coefficient; 95% CI = 95% confidence interval; I² = heterogeneity; p-value = test for subgroup differences.

**Supplemental Table S9** Summary of Findings (GRADE assessment)

| **Outcome** | **N° studies** | **N° implants** | **Effect [95% CI]** | **Risk of Bias** | **Inconsistency** | **Indirectness** | **Imprecision** | **Publication bias** | **Certainty** |
| --- | --- | --- | --- | --- | --- | --- | --- | --- | --- |
| ISQ-IT | 20 | 6160 | 0.44 [0.32 - 0.55] | Serious | Serious | Not serious | Not serious | Undetected | Very low |
| ISQ-Survival | 3 | 769 | 10.22 [-2.14 - 22.58] | Not serious | Serious | Not serious | Serious | Not assessable | Very low |

**Supplemental Table S10** Risk of bias assessment for studies with a retrospective design (NOS).

| **Study** | **Selection (0-4)** | **Comparability (0-2)** | **Outcome (0-3)** | **Overall** | **Risk of bias** |
| --- | --- | --- | --- | --- | --- |
| Turkyilmaz et al., 2008 (BMC) | 4 | 2 | 3 | 9 | Low |
| Degidi et al., 2012 | 3 | 2 | 3 | 8 | Low |
| Levin et al., 2016 | 3 | 2 | 3 | 8 | Low |
| Chen et al., 2019 | 3 | 2 | 3 | 8 | Low |
| Dragonas et al., 2020 | 3 | 2 | 3 | 8 | Low |
| Back et al., 2025 | 4 | 2 | 3 | 9 | Low |
| Kim et al., 2025 | 3 | 1 | 3 | 7 | Low |
| Lombardi et al., 2025 | 4 | 2 | 3 | 9 | Low |
| Tan et al., 2025 | 4 | 2 | 3 | 9 | Low |

N.B. The scores obtained using the NOS were converted into risk of bias categories to facilitate the interpretation of results. Specifically, the NOS scores were classified as follows:
***- Low risk of bias:*** score ≥ 7
***- Moderate risk of bias:*** score 4-6
***- High risk of bias:*** score 0-3

**Supplemental Table S11** Risk of bias assessment of single-arm studies and cross-sectional studies included in the review.

| **Study** | **Checklist JBI** | **Criteria satisfied** | **Risk of bias** |
| --- | --- | --- | --- |
| Fisher et al., 2009 | Cohort Studies | 63.6 % (7/11) | Moderate |
| Degidi et al., 2010 | Analytical Cross Sectional Studies | 75 % (6/8) | Low |
| Park et al., 2012 | Cohort Studies | 90.9 % (10/11) | Low |
| Dias et al., 2014 | Cohort Studies | 72.72 % (8/11) | Moderate |
| Filho et al., 2014 | Analytical Cross Sectional Studies | 75 % (6/8) | Moderate |
| De Santis et al., 2016 | Cohort Studies | 81.8 % (9/11) | Low |
| Norton et al., 2017 | Cohort Studies | 63.6 % (7/11) | Moderate |
| Sarfaraz et al., 2018 | Cohort Studies | 81.8 % (9/11) | Low |
| Park et al., 2019 | Analytical Cross Sectional Studies | 75 % (6/8) | Moderate |
| Rahman et al., 2019 | Cohort Studies | 81.8 % (9/11) | Low |
| Brouwers et al., 2021 | Cohort Studies | 90.9 % (10/11) | Low |
| Do Vale Souza et al., 2021 | Analytical Cross Sectional Studies | 87.5 % (7/8) | Low |
| Noaman and Bede, 2022 | Cohort Studies | 81.8 % (9/11) | Low |
| Rocha Ferreira et al., 2022 | Analytical Cross Sectional Studies | 87.5 % (7/8) | Low |
| Feng et al., 2023 | Analytical Cross Sectional Studies | 75 % (6/8) | Moderate |
| Dkheel et al., 2024 | Analytical Cross Sectional Studies | 75 % (6/8) | Low |
| Ko et al., 2025 | Case Series | 80 % (8/10) | Low |
| Rosen et al., 2025 | Case Series | 100 % (10/10) | Low |

The scores obtained using the Joanna Briggs Institute (JBI) checklist were converted into risk of bias categories to facilitate the interpretation of results. Specifically, the JBI scores were classified as follows:
*Low risk of bias:* score ≥ 70%
*Moderate risk of bias:* score between 50% and 69%
*High risk of bias:* score < 50%
